# What is the impact of structural and cultural factors and interventions within educational settings on promoting positive mental health and preventing poor mental health: a systematic review

**DOI:** 10.1186/s12889-022-12894-7

**Published:** 2022-03-17

**Authors:** David Troy, Joanna Anderson, Patricia E. Jessiman, Patricia N. Albers, Joanna G. Williams, Seamus Sheard, Emma Geijer-Simpson, Liam Spencer, Eileen Kaner, Mark Limmer, Russell Viner, Judi Kidger

**Affiliations:** 1 Bristol Medical School, Population Health Sciences, University of Bristol, Bristol, UK; 2 Department of Psychiatry, University of Cambridge, Cambridge, UK; 3 Medical School, University of Exeter, Exeter, UK; 4 Population Health Sciences Institute, Newcastle University, Newcastle, UK; 5 Faculty of Health and Medicine, Lancaster University, Lancaster, UK; 6 Population, Policy and Practice Department, University College London, London, UK

**Keywords:** Mental health, School environment, Systematic review

## Abstract

Mental health (MH) difficulties are on the increase among children and young people (CYP). Evidence has shown that educational settings contain both risk and protective factors for MH. This review investigated which structural and cultural factors and interventions within educational settings promote positive MH and prevent poor MH in 4–18 year olds. Searches were conducted in PsychINFO, Embase, ERIC, ASSIA and British Education Index, and reference lists from key studies and relevant systematic reviews were hand-searched. Intervention, cohort, and qualitative studies were included. Of the 62 included papers, 36 examined cultural factors (30 social/relational and six value-related) while 12 studies examined structural factors (eight organisational and four physical) and 14 studies examined multiple factors. There was strong evidence for the impact of positive classroom management techniques, access to physical activity, and peer mentoring on student MH. Studies examining the impact of positive school culture, teacher training in MH and parent involvement in school MH activities also found predominantly positive results for student MH, albeit the evidence was of lower quality or from a low number of studies. Few studies explicitly examined the impact of interventions on MH inequalities; those that did indicated limited if any reduction to inequalities. A very small number of studies suggested that interventions targeting those at risk of poor MH due to socioeconomic factors could successfully improve wellbeing and reduce depression, anxiety and behavioural problems. Studies exploring the effect of management and leadership strategies within schools, policies, and aspects of the physical environment other than green space were scarce or absent in the literature. This review highlights the need to consider the ways in which educational settings are organised, the culture that is created and the physical space in order to improve the MH of CYP.

**Supplementary Information:**

The online version contains supplementary material available at 10.1186/s12889-022-12894-7.

## Background

Globally, evidence suggests that mental health (MH) difficulties are on the increase among children and young people (CYP; defined as between 4 and 18 years) over the last 50 years with a trend towards more consistent findings in recent decades reporting an increase in MH difficulties among adolescents especially girls [1–3]. Specific to the UK context, CYP MH difficulties (depression, self-harm) have increased between 2005 and 2015 [4] and probable MH conditions have increased from 2017 to 2020 based on a national CYP MH survey [5]. Concerns regarding the high prevalence of emotional/MH distress among CYP which may not necessarily reach threshold levels for a diagnosable disorder but may underpin higher levels of self-harm in CYP [6], and an awareness of the importance of early intervention, have led to increasing emphasis on preventative community-based MH interventions for this age group. Educational settings, as the place where the majority of CYP spend much of their time, have been highlighted in the UK government’s Green Paper ‘Transforming Children’s Mental Health’ [7] as having a key role to play in prevention of poor MH and early intervention.

This need to support the MH of CYP is likely heightened as a result of the global COVID-19 pandemic and accompanying restrictions. Although it is not clear whether all CYP’s MH has deteriorated more than other age groups, there is evidence that MH has declined among some CYP. A rapid systematic review of the impact of social isolation and loneliness caused by lockdown measures suggests that children and adolescents have been more at risk of depression and anxiety, due to a myriad of factors such as fear of COVID-19 and its effect on family members, social isolation, concern about educational progress, and inability to learn at home [8]. CYP with additional disadvantages such as experiencing economic hardship / parental stress and having special educational needs / neurodevelopmental disorders have been reported to be especially at risk of declining MH during lockdowns [9]. However, the picture is mixed, for example some CYP experienced greater wellbeing and reduced anxiety during the first UK lockdown, possibly due to the removal of stressors within the school environment, such as pressure of academic work, and challenging peer relationships [10]. Many countries have implemented national school closures at various points during the pandemic [11] and it is therefore conceivable that many students, especially those already experiencing MH difficulties pre-pandemic, will have faced considerable challenges adjusting each time they return to a school environment [12], with varying pandemic related restrictions depending on location.

Interventions which focus on changes to the school environment, including physical, cultural, and organisational aspects, are in keeping with an increasing acknowledgement of the importance of considering complex systems when engaging in public health improvement [13] and the need to consider ways to make environments more conducive to better health, including MH [14]. A review completed several years ago of evidence on the impact of the school psychosocial environment on teenage MH [15] yielded five controlled trials, of which three were high quality randomized-controlled trials (RCTs) that examined the impact of a ‘whole school’ approach to MH. None of these approaches led to improvements in MH, possibly due to the challenges of such complex interventions being implemented as planned [16]. Of the 30 cohort studies included in the review, evidence was strongest regarding the importance of good quality relationships with staff. However, very few studies included objective, school-level measures (as opposed to students’ perceptions of their school).

A more recent review looked specifically at the impact of the World Health Organization’s (WHO) health-promoting schools framework on all reported health outcomes [17]. This framework requires intervention at three levels: formal health curriculum, ethos and physical environment, and engagement with families and/or communities. Only three out of 67 included studies measured the impact of interventions on MH outcomes and none found a significant effect. A complementary review considered the impact of school-based interventions that focused on organisation/management, teaching/pastoral care/discipline and the physical environment, on health outcomes [18]. Of the ten studies included in the review, six were RCTs, of which four evaluated interventions that attempted to create school climates characterised by a stronger sense of community and/or better interpersonal relations. There was evidence that these approaches could reduce aggressive behaviour and violence, but only one study, of poor quality, examined MH outcomes, reporting an improvement in social anxiety in elementary schools. A recent review of whole-school universal interventions to promote MH [19, 20] provided an update on earlier reviews on this subject e.g., [16, 21]. Of ten papers that met the inclusion criteria, eight reported those interventions having a positive impact on MH outcomes, but seven studies were of low quality (e.g., lack of comparison groups and small sample sizes) and details on outcomes measures, effect sizes and long-term effects were lacking.

In summary, although there are a number of reviews focusing on MH-related services or support [22–24], there is a paucity of good quality, recent evidence focusing solely on the association between environmental factors within educational settings – organisational, physical, social, and cultural – measured objectively, and CYP’s MH outcomes. Specifically, no previous review has drawn together the evidence to identify which environmental factors in exclusively statutory educational settings are important for promoting good MH, preventing MH problems from occurring and improving MH outcomes when problems do arise. Reviewing such evidence is necessary for developing more effective interventions. This systematic literature review has been conducted to address this need, by asking the following questions:

1. What factors within educational settings influence development of poor MH and/or improvement of MH for CYP?
2. What interventions targeting factors within educational settings are effective at preventing poor MH and/or improving MH in CYP?
3. Are effective interventions targeting factors within educational settings to prevent poor MH and/or improve MH in CYP also cost-effective?
4. Do effective interventions targeting factors within educational settings to prevent poor MH and/or improve MH in CYP contribute to reducing or widening MH inequalities?

## Methods

### Protocol and registration

The protocol for this systematic review was registered with PROSPERO on 17^th^ June 2019 (CRD42019138976) [25].

#### Eligibility criteria

Inclusion/exclusion criteria are outlined in relation to each research question using the PICOS approach [26] (See Table 1).

**Table 1:** Inclusion and exclusion criteria

Criteria	Specification
Population	Include: Studies where the target population were pupils aged 4–18 years
Intervention	Include: Intervention or non-intervention studies that have reported on either 1) the impact of one or more physical, cultural or organisational factors within a statutory educational setting on student MH or 2) the impact of a change to physical, cultural or organisational factors within a statutory educational setting on student MH or 3) the impact of an intervention targeting one or more physical, cultural or organisational factors within an educational setting
	Exclude: Studies where the intervention was MH-related services or support (e.g., counselling), studies that only reported on individual perceptions of environmental factors, or did not report any MH outcomes
Comparator	Include: Non-intervention studies where the comparison group had no exposure to the physical, cultural or organisational environment factor(s) of interest Intervention studies where the comparison group could be those in receipt of a different intervention targeting the same factors, or no intervention at all
Outcomes	Include: Studies that reported on improvements in positive MH; reduction of incidence, prevalence, severity or recurrence of poor MH and/or of self-harm or suicidal thoughts or behaviour
Setting	Include: Studies conducted in statutory education settings. Statutory educational settings are those that provide formal statutory education and includes schools, and non-mainstream settings such as those for CYP who have been excluded from school (pupil referral units (PRUs) in the United Kingdom (UK)) and those for CYP who have special needs. It also includes colleges of further education, although these will also include students older than the age of 18
	Exclude: Studies in non-formal or non-statutory settings such as youth and sports clubs, or nurseries
Study design	Include: Randomised controlled trial, quasi-randomised controlled trial, controlled before-and-after study, prospective cohort study, and qualitative studies. Natural experiments provided they had a valid comparator
	Exclude: Cross-sectional, or case studies of individual pupils. Non-empirical studies such as letters, commentaries, editorials, opinion pieces and book reviews
Country, language	Include: Any country Full text in English

*CYP* children and young, *MH* mental health, *UK* United Kingdom, *PRU* pupil referral units

Environmental factors from a previous review [17] (full details can be found in our protocol [25]) were used to guide the search strategy. This list of environmental factors was not exhaustive and was amended during the screening stage. Two young people’s advisory groups were consulted about factors in the school environment that were most important for MH, which also informed our search terms. Studies reporting on environmental factors not on the list were discussed by the whole research team and a decision made as to whether they were eligible for inclusion. As an example, streaming classes by ability was added. Studies reporting on curriculum-based interventions, and those designed to improve individual knowledge or skills were excluded unless they also included physical, cultural or organisational factors.

### Search strategy

Relevant studies were identified through systematic searches of the following electronic bibliographic databases: PsychINFO, Embase, ERIC, ASSIA and British Education Index. In addition, reference lists from key studies and relevant systematic reviews were hand-searched. The development of search terms was informed by the analysis of titles, abstract and index words of key publications in the field. We used the combination of terms for educational settings, environmental factors and MH outcomes. For an example search strategy see Table 1 in the Supplementary File. Database searches were conducted between 18/06/2019 and 20/06/2019. Search results were managed using EndNote software, and individual database results were merged with duplicates removed. The merged EndNote file was then uploaded into the online software CADIMA (https://www.cadima.info/index.php/area/evidenceSynthesisDatabase), designed to support evidence synthesis.

### Study selection

In the first selection stage titles and abstracts were screened independently by five researchers (DT, PJ, JA, JK, JW), with 10% of titles double screened and inconsistences resolved by discussion with a third researcher. Full-text screening was undertaken independently by four researchers (JA, DT, PJ and JK); 15% were double screened and inconsistencies resolved by discussion with a third researcher.

### Data extraction

Data extraction was conducted by DT, PJ, EGS, LS, SS and PA, and 10% double extracted as a quality and consistency check by JK. The following data were extracted into a spreadsheet:

- Author, year of publication
- Country
- Study title and aims
- MH outcome (e.g., depression, anxiety, wellbeing)
- Environment factor(s) of interest
- Setting type
- Study design
- Sample size (i.e., no. of schools/other education setting/individuals)
- Sample size justification
- Demographic characteristics
- Study exclusion criteria (settings and individuals)
- Response rates (baseline; follow ups)
- Description of the intervention (if relevant) including duration, key components and implementation details were extracted, as was the control group treatment.
- Measures
- Comparator
- Data analysis
- Findings on primary and secondary outcomes (including outcomes not related to MH; only MH-related outcomes were used in the analyses and reporting of findings). Findings from each study were extracted separately for each research question 1–4.

### Quality appraisal

Critical appraisal of the quality of all quantitative studies was undertaken using the Canadian Effective Public Health Project Practice Quality Assessment Tool for Quantitative Studies (http://www.ephpp.ca/PDF/Quality%20Assessment%20Tool_2010_2.pdf). This tool was developed to assess the quality of quantitative studies including RCTs, before-and-after studies and case–control studies [27]. The tool doesn’t assess the risk of biased results but rather gives each study a quality rating of strong, moderate or weak. For qualitative studies, we used the Critical Appraisal Skills Programme qualitative checklist which includes two screening questions (aims of the study and appropriateness of qualitative methodology to the aims), and a further eight appraisal items (research design, recruitment strategy, data collection, reflexivity-related issues, ethical issues, rigour of analysis, and the reporting and value of findings).

Each study was critically appraised independently by two members of the research team (DT, PJ, EGS, LS, SS and PA). Ratings were compared, and where there was disagreement, a third appraisal was undertaken by JK. No studies were excluded on the basis of quality appraisal, but quality is considered in the narrative summary of findings.

## Results

### Overview of included studies

**Fig. 1:**
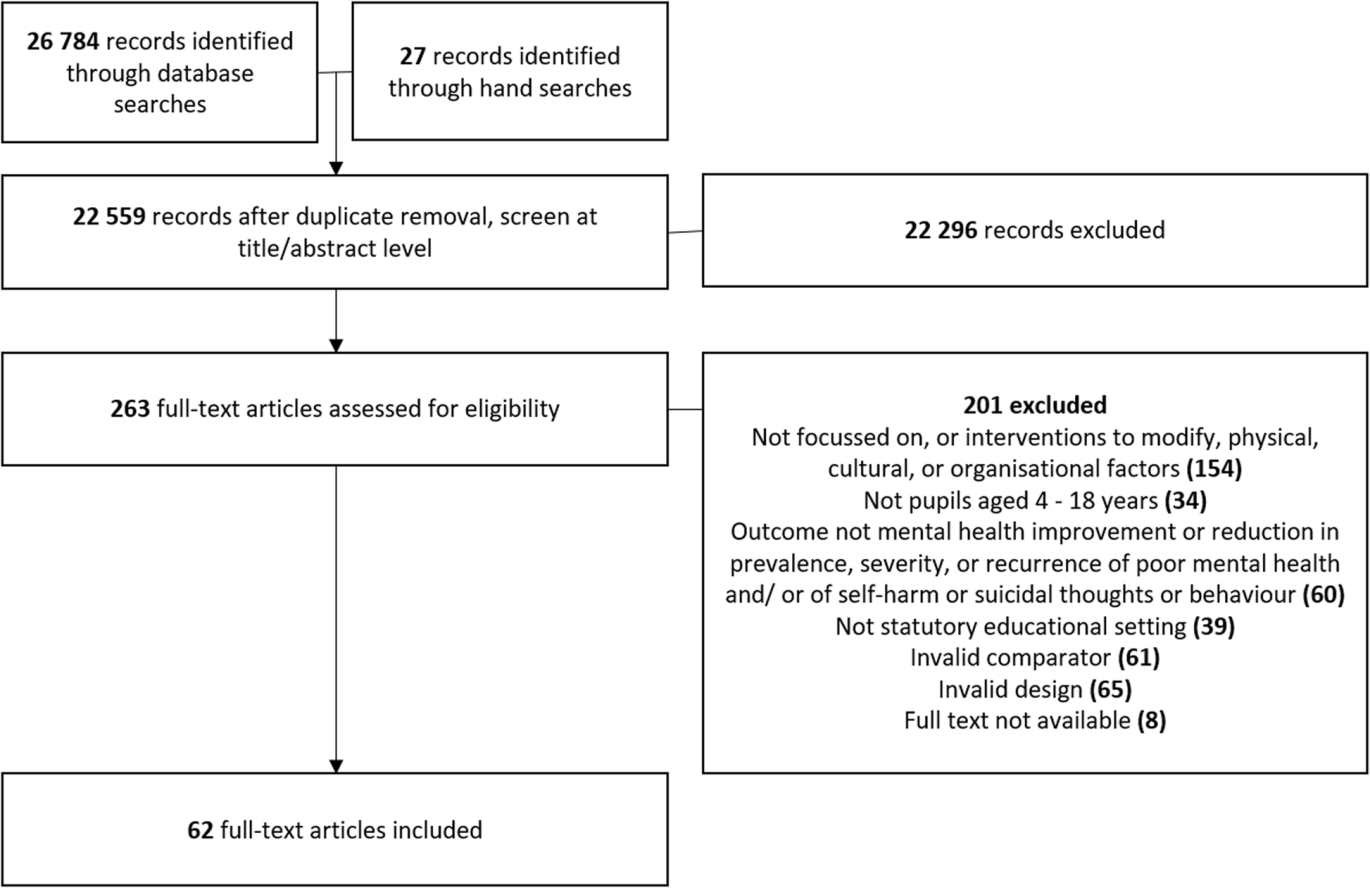
Flowchart of the study selection process

A flowchart showing the study selection process is shown in Fig. 1 below.

A summary of the characteristics of included studies can be found in Table 2. A majority were published after 2010. Studies were based in a range of countries, with the United States of America (USA) being the most common country (24 studies), followed by the UK (12). There were slightly more studies based in primary than secondary school, with 3 based in pupil referral units (where students have been excluded from mainstream school.

**Table 2:** Characteristics of included studies

Criterion	Characteristic	No of studies (total *n* = 62)
Year	Before 1990	1
	1991–2000	3
	2001–2010	17
	After 2010	41
Country	USA	24
	UK	12
	Australia	6
	Canada	5
	The Netherlands	3
	Finland	2
	Other (single countries)	10
Setting	Primary school	20
	Secondary school	29
	Both primary & secondary	9
	Pupil referral units	3
	Not stated	1
Study design	RCT	25
	Controlled trial	11
	Qualitative	11
	Cohort analytic	8
	Mixed methods	6
	Case control	1

*RCT* Randomised Controlled Trial, *USA* United States of America, *UK* United Kingdom

### Quality of included studies

#### Quantitative studies

Of the 52 papers assessed, there was an almost even distribution of those with an overall quality rating of strong (28.8%), moderate (38.5%), and weak (32.7%). A total of 25 papers were RCTs, of which 8 (32%) received a strong overall rating. Data collection methods were moderately strong across all papers, with 60% of papers receiving a strong rating for this section. This is explained by the high percentages of papers reporting the use of valid and reliable tools (81.1% and 69.8%, respectively). Ratings for individual studies can be found in Table 2 in the Supplementary File.

#### Qualitative studies

All qualitative papers had a clear statement of research aims and qualitative methodology was considered appropriate. However, a lack of reporting on recruitment strategy and data analysis methods was common and made assessment difficult. All studies were deemed to be of limited or moderate research value. Assessments for individual studies can be found in Table 3 in the Supplementary file.

### Findings

#### Q1 & 2. Findings on what factors within educational settings influence development of poor MH and/or improvement of MH for CYP and what interventions targeting factors within educational settings are effective at preventing poor MH and/or improving MH in CYP

**Fig. 2:**
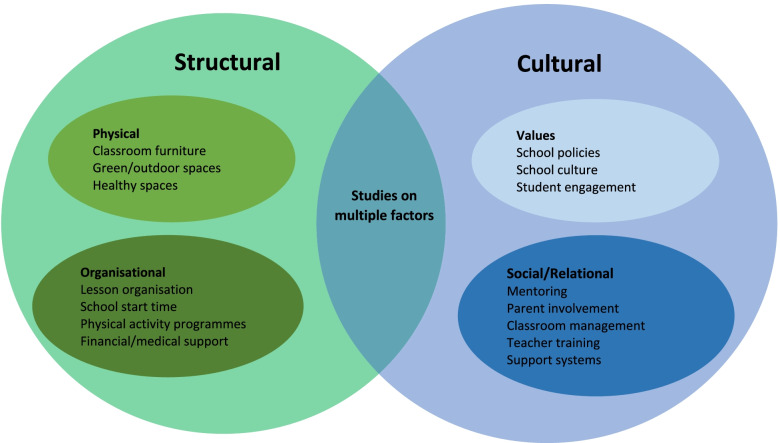
Grouping of studies

In summarizing findings for Q1 and Q2, we have organised the included studies into those that focused on structural factors, those that focused on cultural factors, and those that covered more than one type of factor (Fig. 2). We defined structural factors as recurrent patterned arrangements which influence or limit the choices and opportunities available within the setting. Within structural factors are *organisational factors* (i.e., the way life in the educational setting operates including school rules and how both learning and free time are organised, activities/supports that are offered, how communication with parents is organised, etc.) and *physical factors* (all aspects of the physical environment, including layout of classrooms, equipment, quality of buildings and how space is used). We defined cultural factors as the values promoted within a setting and how individuals treated one another (e.g., inclusivity, kindness, equality, hard work, quality of relationships/styles of interaction, actions taken/policies to give students a voice, encourage feelings of belonging, feeling valued, helping each other, etc.). This was further divided into values and social/relational factors.

Sixteen studies reported on Q1 (factors within educational environments and their impact on MH); ten qualitative studies and six quantitative studies. Forty-six studies reported on Q2 (interventions to change a factor within educational environments and impact on MH); 39 quantitative studies, six mixed methods studies and one qualitative study.

**Fig. 3:**
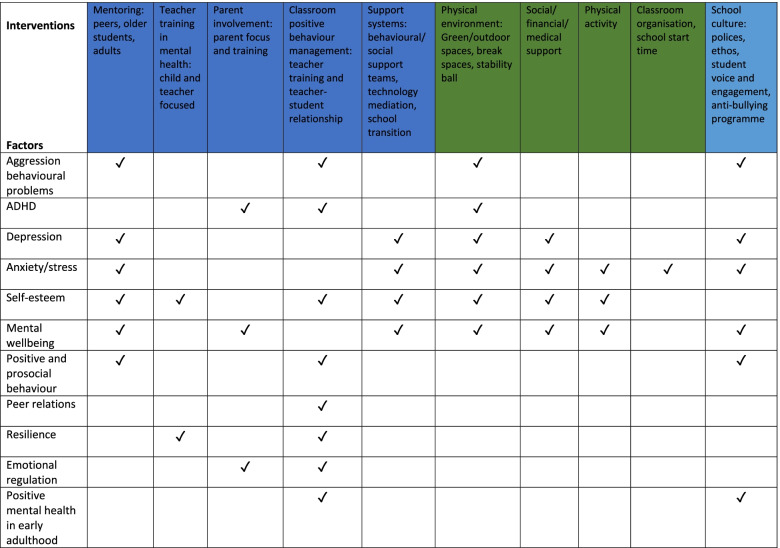
Map of relationships between interventions/factors and outcomes

A model summarising findings regarding the relationships between different environmental interventions/factors and outcomes is represented in Fig. 3. A summary of the findings from each study can be found in Table 4 in the Supplementary file.

## Structural

### Organisational

#### Opportunity to engage in physical activity

Evidence from five studies suggests that where educational settings provide opportunities to engage in physical activity this has a positive impact on students’ MH and wellbeing. Improvements were seen in physical self-worth, self-perceived sport competence, body attractiveness, social competence and global self-worth [28], stress and self-rated MH [29], anxiety [30] and anxiety and self-esteem [31]. Two studies also reported improvement in depression [29, 32] while two [30, 31] found no change to this outcome.

#### Organisation of lessons

Three related cohort studies with longitudinal designs across 161 secondary schools examined maths streaming, and found that individual achievement produces positive emotions (regardless of set), but that when an individual is in a high achieving set, this reduces positive emotions by reducing self-confidence, enjoyment, and increasing negative emotions such as anxiety and hopelessness [33].

#### School start time

One RCT study found no difference in happiness, depression and suicidal thoughts/plans between treatment and control groups when school start time was delayed by at least 30 min, although these outcomes improved across both groups [34], while another mixed methods study found a later start time led to improved subjective health and reduced stress [35].

#### Financial and medical support

School supports consisting of payment of school tuition fees, provision of a school uniform, and monthly nurse visits marginally reduced depression symptoms among orphans in Kenya by Year 4 of the intervention [36].

### Physical

Evidence from two papers suggested that green and outdoor space were associated with reduced problem behaviours, aggression, stress, Attention Deficit Hyperactivity Disorder (ADHD) and depressive symptoms [37] and improved mental, emotional and social health [38]. Sitting on a stability ball compared to a regular classroom chair resulted in a reduction in depressive/anxious symptomatology after eight weeks but not at a five month follow up [39]. Healthy spaces created both inside and outside of the school buildings – in which students and teachers could enjoy healthy snacks, movement breaks, and MH breaks—was associated with an initial decrease in depression and anxiety but these improvements were note sustained at follow up [40].

### Cultural

#### Values

#### School ethos

Poor school ethos (defined as poor quality pupil-teacher relationships, poor quality physical environment, and pupils feeling disengaged and not part of the school) as assessed by a research nurse, was associated with risk of self-harm at 19 but not once adjusted for confounders [41] and a whole-school anti-bullying intervention reduced levels of anxiety [42].

#### Student voice

*“Got health?”* teams made up of students, staff and community partners using action research to create a more positive school climate reported positive changes to school culture relating to MH perception, awareness and stigmatisation [43].

#### School policies

Policies fostering equality and inclusion and acknowledging cultural pluralism promoted better well-being and fewer psychological and behavioural problems [44]. The introduction of healthy school policies and a healthy school environment involving parents, teachers, and neighbourhood did not reduce psychosocial problems [45] but did decrease depressive symptomology [46].

### Social/relational

#### Classroom management techniques

Classroom management techniques involving positive behaviour management approaches, teacher training and a focus on improving teacher-student relationships had generally positive effects on a range of outcomes: improved prosocial behaviour and fewer problems with peers [47], a reduction in disruptive behaviours, and improved prosocial behaviours and emotion regulation [48], decreased inappropriate behaviours and increased positive behaviours [49], a reduced risk of future suicide attempts [50], improved positive functioning in school and at work and reduced emotional and MH problems at 21 years of age [51], increased levels of resilience, more positive behaviours and fewer challenging behaviours [52], reduced disruptive behaviour [53], reduced psychosocial problems at nine months but not at 18 or 30 months follow up [54] and better social competence and emotional self-regulation and fewer conduct problems [55].

#### Mentoring (peer)

Peer mentoring interventions, some of which involved older students from other settings mentoring younger students, had mixed effects on MH across different studies. Improvements in behaviour were reported by four studies [56–59], while two studies reported no change [60, 61], A reduction in depression was reported in one study [57] while two found no effect for this outcome [60,61]. Anxiety was reduced in two studies [60, 62] but not in another [63], while wellbeing, happiness, and positive MH were improved in three studies [57, 59,59, 64]. Social skills and relationships with others were improved in one study [58] but not in two others [60, 61]. Self-esteem was improved in one study [58] but not in two others [62, 63]. An additional study investigated the effects of mentoring on the mentors’ MH and reported improved emotional intelligence and self-worth [65].

#### Mentoring (adult)

Adult mentoring interventions improved mental wellbeing and self-efficacy in one study [66], and increased self-esteem among high school students at risk of experiencing emotional or behavioural difficulties in another [67]. However, a third study reported no change in social and emotional health in at-risk students (defined as such due to minority ethnic status, low socioeconomic status (SES) or poor academic history) [68].

#### Involving parents in school MH activity

Two studies that delivered interventions focused on training parents in child coping skills, behaviour management and family cohesion found improved depression, self-regulation and school engagement [69], and improved internalising but not externalising symptoms among low-income Mexican American students [70].

#### Teacher training in MH

Teacher training in promoting social, emotional and MH of students improved resilience and self-esteem in students with emotional difficulties [71]. However, teacher training in MH first aid found no effect on student psychosocial problems [72].

#### Support systems

Behavioural, emotional and social support systems improved self-esteem, confidence and overall happiness [73] and reduced depression but not anxiety, loneliness, and face-to-face victimization [74]. In one study, support for students transitioning from primary to secondary education improved depression, anxiety, stress, feelings of loneliness and perceptions of school safety at the end of the students’ first year in secondary school, but none of these differences were sustained [75]. In another study, such support lowered school anxiety but not generalised anxiety when controlling for prior anxiety [76].

## Studies exploring multiple factors

One group of four studies combined teacher training in support/classroom management with improving parenting skills, and found a reduction of ADHD symptom severity [77, 78] lowered conduct problems, improved social problem solving [79], and improved conduct disorder, oppositional defiant disorder, ADHD and anti-social behaviour [80].

A group of qualitative studies explored a wide range of aspects of the educational environment in relation to MH outcomes. Three studies in PRUs identified positive teacher/student relationships and peer relationships, a calm, personalised learning environment, less crowded classrooms and effective disciplinary sanctions as important for reducing disruptive behaviours [81, 82] and improving behaviour [83]. A primary school-based study identified outside space, a clean environment, positive relationships with others, social and emotional supports and extra-curricular activities as important for self-esteem [84], and a study in secondary schools identified relationships with teachers and peers, a positive culture relating to MH, appealing physical environments, access to support and access to safe spaces as important for student emotional health and reducing distress [15].

A group of five studies reported the impact of whole school interventions involving multiple components such as building a supportive culture and environment, providing support services, involving students in school decision making and involving parents or families in school activities found differing effects on MH outcomes. Only one such study reported positive effects on improvements to quality of life, wellbeing and psychological problems [85]. Of the others, three found no impact on depression [40, 86, 87], one reported no improvement in anxiety [40], and one reported no differences in total difficulties, internalising problems, and prosocial behaviour, although for those with high baseline scores there was a differential effect in favour of the *control* group [88]. An additional study that took a similarly holistic approach but was targeted specifically at teenage mothers reported improvements in self-esteem and positive emotional health [89].

### Q3. Findings on cost-effectiveness of interventions targeting factors within educational settings.

Only three studies, all of which had reported interventions to be effective, included information about cost effectiveness. Two of these clearly reported a formal cost effectiveness evaluation. The INCLUSIVE cluster randomised controlled study in UK secondary schools [85] reported what was considered a low additional cost of £47-£58 per student over a two year intervention period, which was found to be effective at improving MH outcomes. The STARS cluster randomised controlled study in UK primary schools [54] looked at total economic costs after 30 months including service provision, and found a cost-effectiveness ratio favouring the intervention of approximately -£29.70 per unit improvement on the main outcome measure (Strengths and Difficulties Questionnaire). The final study, the Fast Track randomised controlled trial, aimed to reduce conduct disorders and antisocial behaviour among children identified as ‘high risk’. The authors estimated an intervention cost of $58,000 per young person over 10 years but reported this as potential economic saving compared to the much larger cost to society per young person who commits a crime. However, the authors note as the intervention was only found to be effective for those identified as highest risk, it may only be cost effective if directed to the highest risk group [80].

### Q4. Findings on the impact of interventions targeting factors within educational settings on MH inequalities.

Most included studies did not explicitly look at the impact of the environmental factors or interventions on reducing or widening inequalities. Six studies specifically targeted CYP who were at risk of poor MH due to socioeconomic circumstances, which, although not providing formal measures of impact on inequalities, do highlight interventions with the potential to prevent or reduce poor MH among those at greatest risk. The wide range of interventions led to mixed results. One study found older peer mentoring did not improve socio-emotional health among economically disadvantaged young people compared to less disadvantaged controls although positive trends were noted [68]. One study reported improvements in anxiety and self-esteem scores but not depression among young people in a low SES area participating in a physical activity programme compared to controls [31]. One study found perceived manifestation of diversity policies improved psychological school adjustment among young immigrants [44]. One study found a multifactor intervention (parent training, child mentoring, home visits and parent–child groups), delivered in schools in areas high in crime and poverty, showed reductions in behavioural problems, with greater improvements among students with higher conduct problems [80]. One study found a culturally sensitive mentoring programme for minority ethnic young people resulted in improved positive MH [64]. One study reported that delivering a yoga intervention to CYP from disadvantaged socioeconomic backgrounds reduced anxiety and stress but not depression overall [30]. Finally, one study reported marginally reduced depression symptoms when financial and health support was offered to orphans at school in Kenya [36].

Of those studies that specifically conducted subgroup analyses, five looked at differences in effect for those at higher risk of poor MH due to social or economic factors, compared to those not at risk. None of the studies provided evidence of an intervention reducing inequalities. One study did not find that their group based mentoring intervention had a greater impact on the MH of CYP who had special educational needs or who had failed at least two classes, although they did see a decline among the at-risk group for absence and slight improvements in life skills (e.g., problem-solving) compared to those not at risk [67]. Similarly, another study found no difference of effect for their school-wide positive behaviour intervention on children with special educational needs compared to those without [48]. One study also found no difference in outcomes by socioeconomic group for their multifactor intervention [85]. One study reported that, following a physical activity intervention, disparities in physical self-worth between those with higher versus lower SES widened among the control group, whereas they remained constant among the intervention group, with everyone’s self-worth improving [28]. Finally, one study found no difference in their mentoring programme on MH outcomes by ethnicity [59].

In total, 13 studies examined differences by sex in their subgroup analyses, of which 8 found no differences in MH outcomes by sex [36, 48, 59, 61–63, 69, 86]. Two studies reported differences by sex that may have indicated a widening of inequalities. One of them found their multi factor intervention reduced bullying and aggression among early adolescents and had a bigger impact on boys’ MH (SDQ total difficulties score; boys -1.29, 95% CI -1.67, -0.92; girls 0.04, 95% CI -0.30, 0.39, *p* < 0.0001) and wellbeing outcomes (Short Warwick-Edinburgh Mental Well-Being Scale total wellbeing index; boys 1.32, 95% CI 0.89, 1.74; girls 0.04, 95% CI -0.81, -0.04, *p* < 0.0001; Pediatric Quality of Life Inventory; boys 3.85, 95% CI 2.89, 4.80; girls -0.41, 95% CI -1.28, 0.46, *p* < 0.0001). As girls tend to have poorer MH and wellbeing in the teenage years it is possible this finding signals an increase in inequality, but this was not explicitly discussed by the authors [85]. The other study reported a bigger reduction in girls’ conduct (SDQ conduct: *B* -0.07, *p* < 0.001) and peer problems (SDQ peer: *B* -0.05, *p* < 0.001) and a bigger increase in their prosocial behaviours (SDQ prosocial: *B* 0.19, *p* < 0.001), with no sex differences in emotional problems, following a whole school positive behaviour intervention in primary schools. Boys tend to be at increased risk of behavioural problems within this age group, therefore this may indicate the intervention widened inequalities by sex, but again this was not discussed [47]. On the other hand, two studies reported findings that may have indicated a reduction in MH inequalities, or at least prevention of them widening. One of them found boys had higher physical self-worth at baseline (Children’s Physical Self-Perception Profile: *p* < 0.001), but that following a physical activity intervention, boys and girls in the intervention group improved equally in physical self-worth, whereas this gap by sex widened in the control group (*p* < 0.05) [28]. The other study in a post hoc analysis report their mentoring programme led to a bigger improvement in positive MH for girls (Mental Health Continuum – Short Form: *b* = 11.23, *p* = 0.037). Although they do not report baseline differences by sex, girls generally have poorer wellbeing, pre-intervention [64]. Finally, one study reported a greater reduction in depression among boys than girls, but unusually, boys began with higher baseline depression (three depression items adapted from SDQ; pretest: boys = 1.45, girls = 1.29), therefore in this study MH inequality by sex remained but switched from boys having higher depression to girls having higher depression (posttest: boys = 1.30, girls = 1.23) [30].

## Discussion

Findings from our systematic review of 62 papers provided evidence as to which school-based interventions, and factors are effective in preventing poor MH and promoting positive MH. The greatest amount of evidence existed in relation to the importance of supportive relationships. Specifically, classroom management techniques in the included studies and peer mentoring schemes had impacts on both positive MH outcomes (such as self-esteem, happiness, and prosocial behaviours) and negative ones (e.g., reductions in aggressive behaviours, depression, and anxiety), although some studies found no change in some of the outcomes considered. Studies examining aspects of school culture, and opportunities to engage in physical activity, also reported effects on MH improvement and prevention of poor MH. Parent involvement in school MH activities, teacher training in MH, and provision of support systems were found to have some positive effects, albeit the evidence was of lower quality or from a low number of studies. In other words, we found evidence that organisational, physical, social/relational, and value-related aspects of educational environments were all important for student MH. Evidence regarding the potential impact of such features for MH inequalities was limited. Those studies that looked at a differential intervention effect by sex found no difference, or contradictory or unclear findings. Those that looked at whether interventions had greater impact among those facing socioeconomic disadvantage or from minority ethnic groups found no evidence for this. A handful of studies targeted at risk groups only and reported some positive effects for interventions that focused on physical activity, diversity and inclusion policies, mentoring, yoga and financial / health support.

### Cultural factors (relational and values)

The importance of supportive relationships for MH in educational settings has been reported previously. A previous review that found that successful peer support schemes reported positive outcomes such as increased happiness or wellbeing, improved self-esteem, and confidence. Successful schemes were well run, had a clear focus, good coordinators and received support from throughout the school including from senior school management [90]. This highlights the importance of ensuring discreet interventions are fully embedded within school life to have the most success. Two of our three included studies found mentoring by adults improved MH [66, 67]; other research on school-based adult mentoring found positive effects on prosocial outcomes for at risk youth, but this was dependent on the quality of relationship developed between mentor and mentee [91].

The nine studies that looked at classroom management techniques found predominantly positive results [47–55]. Techniques broadly focusing on positive behaviour supports, incentives for better behaviour and clear consequences for undesirable behaviour were effective in reducing disruptive behaviours and increasing prosocial behaviours. A review consisting of nine RCTs investigating the effectiveness of one particular classroom management programme—the Incredible Years intervention—reported reduced use of negative classroom management strategies among teachers and reduced conduct problems among high-risk children but no effect on improving prosocial behaviours [92]. These different findings could be due to the extent to which the various interventions focused specifically on prosocial behaviours. Of the nine studies in our review, only one involved students of secondary / high school age [53], despite evidence that good quality relationships with teachers are important for MH among teenagers too [93]. The positive impact reported by Närhi et al. on positive impact on disruptive behaviour, as well as on concentration for learning and time teachers reported spending on behaviour management, highlights the potential value of introducing positive behaviour management programmes into secondary as well as primary settings [53].

Studies that focused on values within secondary school settings (e.g., evaluating school ethos [41], involving student voice to improve school climate [43], changing values through school policies [44]) were largely effective in terms of the number of studies showing improvement in MH outcomes, although the type of study designs and their quality were quite varied. Three qualitative studies in different settings (two in PRUs [81, 83], one in mainstream secondary schools [15]) found that good relationships between staff and students and amongst peers were conducive to positive MH outcomes. This is supported by other reviews in this area that have emphasised the importance of good relationships in school climates and school connectedness, as well as students’ sense of school safety and the academic environment in the promotion of good MH [94, 95].

### Structural factors (organisational and physical)

Fewer studies in our review considered the association between organisational aspects of school life and student MH. Interventions which focused on increasing physical activity in secondary schools were broadly effective at improving MH [28–31], as reported by others [96]. A review of reviews in school and non-school settings found positive associations between physical activity and improvements in depression, anxiety and self-esteem in children and adolescents. However, there was only a partial case for causality for reductions in depression and no case based on current evidence for improvements in anxiety and self-esteem. Severity of MH symptoms and physical activity intensity, frequency and duration appeared to be important variables in the success of interventions [97]. Further research is needed to accurately tailor physical activity interventions to optimise MH gains in young people. Future studies should focus on assessing physical activity interventions in primary schools to establish what works with regard to improving the MH of children early in their lives. There were very few studies that examined the impact of other aspects of school organisation, for example there were no studies examining the impact of policies relating to behaviour, or the way the curriculum and break times were organised.

Four studies focused on altering the school physical environment [37–40]. There was some evidence that providing children with access to outdoor, green areas had a beneficial effect on their MH [37, 38] which is in line with other work in this area showing the positive effect on children’s and adolescent’s MH of green space exposure [98]. Exposure to nature in school settings whether it be passively experiencing enhanced green space in the school environment [99] or actively participating in lessons in nature [100] appear to improve child wellbeing. Broadly, evidence suggests that nature exposure is part of a ‘balanced diet’ of childhood experiences that supports healthy child development and wellbeing [101]. More research into how we can incorporate the knowledge of how nature can benefit MH into the school environment is needed. Evidence regarding other aspects of the physical environment of schools was limited to two studies that examined use of stability balls [39] and creation of ‘healthy spaces’ [40]. There were no studies exploring the impact of the condition of buildings, or schools’ layout or facilities.

### Strengths and limitations

This is the first systematic review of the evidence regarding the impact of all structural and cultural aspects of educational environments on the MH and wellbeing outcomes of CYP. A strength of our review is the rigour and transparency of the review process evidenced by the publication of the review protocol prior to the selection and screening of studies. The population, intervention, outcome, and comparator of interest were clearly stated as well as our exclusion and inclusion criteria. Our review included quantitative and qualitative studies which allowed us to summarise the evidence from a broader range of studies and triangulate our findings. By only including studies that examined objective changes or features of the environment, we avoided the potential for reporting bias inherent in self-report measures. Further, our review offered an international perspective from 17 countries, however these were concentrated in higher income countries, and our findings may not generalise to low and middle income settings.

Limitations included the limited number of quantitative studies that were high quality; 70% scored a moderate or weak quality rating. Common methodological shortcomings were failure to blind participants to intervention condition and biases in selecting participants. The quality of qualitative studies was also variable with a lack of reporting on recruitment strategies and data analysis methods quite common. Studies with long-term follow up were also in the minority in this review. Less than half of the included studies conducted subgroup analyses by socioeconomic factors to enable evaluation of the effect of interventions or environmental factors on MH inequalities, and the number of studies that evaluated the cost effectiveness of interventions was low (3 studies) [54, 80, 85]. As this review included studies predominantly published in peer-reviewed journal, publication bias may skew findings in favour of positive/effective outcomes. The review included papers in the English language exclusively, excluding relevant studies in other languages. Finally, only 10% of abstracts and 15% of full texts were double-screened, and although we achieved high inter-rater agreement (ƙ = 0.91 and ƙ = 0.93 respectively), it is possible that some papers that fulfilled inclusion criteria might have been missed.

### Gaps in the literature

Some of the factors within educational environments likely to be important for student MH outcomes did not feature in our review, or only featured in one or two studies. This is likely because many studies exploring aspects of school life do not explicitly look at the impact on student MH. Notably, evidence regarding the impact of school leadership and management strategies was lacking, and there were only three included studies assessing the impact of school policies on student MH [44–46]. The impact of these aspects of governance on MH should be explored in future studies; the downstream effects of leadership and policy changes on student MH have the potential to be substantial given their role in establishing and maintaining rules, processes, and a particular culture within schools.

Only two studies in our review looked at the impact of training teachers in MH support [71, 72]. Concern over a lack of training and support for teachers as part of a school’s overall approach to MH support was highlighted in a survey of over 600 UK schools [102]. A more recent survey also showed low levels of teacher training in MH, with 12% of teachers surveyed having received MH first aid training and 11% received training on common MH conditions [103]. In England, there has been recent commitment to providing training in MH support in secondary schools, creating MH champions, and creating better link up with Child and Adolescent Mental Health Services. Mental Health Teams will also be funded who will support schools to audit aspects of how schools prevent poor MH and promote wellbeing [104, 105]. Teachers involved in these initiatives may be able to act as strategic leads for implementing MH interventions and focus on making organisational and cultural aspects of school life more supportive of MH. These schemes also have the potential to allow cross-learning between these two groups of professionals to raise the standard of MH support in schools. It remains to be seen if these schemes will have a positive effect on organisational and cultural aspects of school life, and ultimately on student MH outcomes. In addition, lack of support for teachers’ own MH remains a concern, with a recent survey (October 2020) of education professionals across the UK showing low rates of wellbeing compared to the general population, and high workload the main reason given by those who were considering leaving the profession [106]. These issues may be currently exacerbated due to extra responsibilities in making schools COVID-19 safe and providing additional support for students whose MH or learning has suffered during the pandemic.

Studies exploring the impact of student voice on MH were lacking, with only one included in our review [43]. The co-development of interventions with student involvement should become standard practice in order to ensure a student-centred and inclusive approach [84, 107]. The impact of the physical school environment on student MH is also an under researched area in school-based research, despite the likely implications for student wellbeing and safety. Human Scale Education is a movement interested in the re-design of schools’ physical space to be more relationship focused (https://www.humanscaleeducation.com/our-principles), but the evidence base for this is currently lacking.

Studies set in further education (FE) colleges (i.e., educational settings that provide technical and professional education and training for young people typically aged between 15–18), were absent from the review. Given that young people with learning difficulties, those from poorer backgrounds and those who struggled to engage with school are overrepresented in FE colleges, and these groups are also at risk of poorer MH, increasing the evidence base regarding MH supporting environments in this setting would be an opportunity to reduce MH inequalities. Finally, evidence of the impact of involving parents in school life seems to be limited to training programmes on behaviour management and parenting skills, which generally took place away from the school environment (i.e., taking place in the parent’s home). Future studies could better integrate parents into interventions taking place in school to ensure a joined-up approach that could enhance the impact on their children’s MH.

## Conclusions

Our review demonstrates that support for CYP’s MH does not only have to come from discrete, classroom-based interventions, but in fact educational settings can support MH by focusing on the culture that is created, the quality of relationships, the way in which life is organised, and the spaces and activities to which students have access. Although we report evidence of positive impact in both improving mental wellbeing and preventing MH problems across these different domains of educational environments, we also identified gaps where evidence is limited or missing. We also found that studies need to evaluate the differential impacts of environmental interventions and features, to ensure MH inequalities are reduced, and the cost effectiveness of interventions or changes.

It is important to acknowledge that schools and other educational settings cannot be a panacea to prevent or treat all MH problems in CYP. However, education leaders and policy makers can consider the ways in which day to day life in these settings can be changed to foster good MH and resilience in all students and provide early support for those experiencing difficulty. COVID-19 and related disruptions to school life has created both a risk to young people’s MH, but also an opportunity to re-evaluate all aspects of the school environment and identify areas in which policies, processes and norms can be changed for the better.

## Supplementary Information

**Additional file 1.**

## Data Availability

All data generated or analysed during this study are included in this published article and its supplementary information files.

## Abbreviations

MH: Mental health
CYP: Children and young people
RCTs: Randomized-controlled trials
UK: United Kingdom
USA: United States of America
ADHD: Attention deficit hyperactivity disorder
SES: Socioeconomic status
PRUs: Pupil referral units
FE: Further education
