# Supplementary material for: What is the impact of structural and cultural factors and interventions within educational settings on promoting positive mental health and preventing poor mental health: a systematic review
Source: BMC Public Health. 2022 Mar 17;22:524. doi: 10.1186/s12889-022-12894-7 (PMC8927746; doi:10.1186/s12889-022-12894-7)
Supplement: Supplementary file 1 — Additional file 1. [file 12889_2022_12894_MOESM1_ESM.docx]

**Table 1: Search strategy**

| Search string | Database |
| --- | --- |
| ((((((((((((schools[MeSH Terms]) OR students[MeSH Terms]) OR ((primary[Title/Abstract] OR secondary[Title/Abstract] OR middle[Title/Abstract] OR high[Title/Abstract] OR junior[Title/Abstract] OR infant[Title/Abstract] OR preparatory[Title/Abstract] OR elementary special[Title/Abstract] OR alternative[Title/Abstract] OR grammar[Title/Abstract] OR comprehensive[Title/Abstract] OR vocational[Title/Abstract] OR technical[Title/Abstract] OR secondary[Title/Abstract] OR non-mainstream[Title/Abstract] OR ""non mainstream""[Title/Abstract] OR short-stay[Title/Abstract] OR ""short stay"")[Title/Abstract] AND school*[Title/Abstract])) OR ((""6th form""[Title/Abstract] OR ""sixth form""[Title/Abstract] OR vocational[Title/Abstract] OR technical)[Title/Abstract] AND college*[Title/Abstract])) OR ((technical[Title/Abstract] OR professional[Title/Abstract] OR collegiate)[Title/Abstract] AND institute[Title/Abstract])) OR (prep-school*[Title/Abstract] OR academ*[Title/Abstract] OR kindergarten[Title/Abstract] OR foundation[Title/Abstract] OR reception[Title/Abstract] OR lyceum[Title/Abstract])) OR ((referral[Title/Abstract] OR re-integration)[Title/Abstract] AND unit[Title/Abstract])) OR ((alternative[Title/Abstract] OR specialist)[Title/Abstract] AND provision[Title/Abstract])) OR (""further education""[Title/Abstract] OR student*[Title/Abstract] OR pupil*[Title/Abstract]))) AND (((((((((((((school N2 (environment*[Title/Abstract] OR facilit*[Title/Abstract] OR building*[Title/Abstract] OR architecture[Title/Abstract] OR design)[Title/Abstract] OR ""physical environment""[Title/Abstract])) OR ((outside[Title/Abstract] OR inside)[Title/Abstract] AND space[Title/Abstract])) OR ((psychosocial[Title/Abstract] OR psycho-social)[Title/Abstract] AND environment[Title/Abstract])) OR (organi?ation*[Title/Abstract] AND (culture[Title/Abstract] OR climate)[Title/Abstract])) OR (ethos[Title/Abstract] OR polic*[Title/Abstract] OR leader*[Title/Abstract] OR governance[Title/Abstract] OR schoolyard[Title/Abstract] OR playground[Title/Abstract] OR non-curricul*[Title/Abstract] OR extra-curricul*[Title/Abstract] OR ""after school""[Title/Abstract] OR harass*[Title/Abstract] OR bullying[Title/Abstract] OR racis*[Title/Abstract] OR ""racial abuse""[Title/Abstract] OR discrimination[Title/Abstract] OR safe*[Title/Abstract] OR welcoming[Title/Abstract] OR inclusive[Title/Abstract] OR respect*[Title/Abstract] OR relation*[Title/Abstract] OR belong*[Title/Abstract] OR connected*[Title/Abstract] OR participat*[Title/Abstract] OR ""classroom management""[Title/Abstract] OR pastoral[Title/Abstract] OR ""whole school""[Title/Abstract] OR school-wide[Title/Abstract] OR support[Title/Abstract] OR counse?*or homework[Title/Abstract] OR workload[Title/Abstract] OR exam*[Title/Abstract] OR test*[Title/Abstract] OR assessment*[Title/Abstract] OR transition*[Title/Abstract] OR playtime[Title/Abstract] OR recess[Title/Abstract] OR ""lunch break*""[Title/Abstract] OR holiday*[Title/Abstract] OR vacation*[Title/Abstract] OR ""school day""[Title/Abstract] OR timetable[Title/Abstract] OR ""free period""[Title/Abstract])) OR ((student*[Title/Abstract] OR pupil*)[Title/Abstract] AND voice[Title/Abstract])) OR ((wellbeing[Title/Abstract] OR well-being[Title/Abstract] OR health)[Title/Abstract] AND (service*[Title/Abstract] OR team)[Title/Abstract])) OR ((community[Title/Abstract] OR parent*[Title/Abstract] OR famil*)[Title/Abstract] AND (engage*[Title/Abstract] OR outreach)[Title/Abstract])) OR ((term[Title/Abstract] OR semester)[Title/Abstract] AND (time[Title/Abstract] OR length[Title/Abstract] OR dates[Title/Abstract] OR break*)[Title/Abstract])) OR ((break[Title/Abstract] OR lunch[Title/Abstract] OR play)[Title/Abstract] AND time[Title/Abstract])) OR ((lesson[Title/Abstract] OR class[Title/Abstract] OR period)[Title/Abstract] AND (length[Title/Abstract] OR time)[Title/Abstract])) OR ""School Health Services""[Mesh])) AND #52) AND (((""mental health""[Title/Abstract] AND (prevent*[Title/Abstract] OR promot*[Title/Abstract] OR improv*)[Title/Abstract])) OR ((""prevention and control"" [Subheading] OR ""Secondary Prevention""[Mesh] OR ""Primary Prevention""[Mesh]) OR ( ""Health Promotion""[Mesh] OR ""School Health Services""[Mesh] ))) | PubMed |

**Table 2: Quality Assessment of Quantitative Studies**

| **1st author (year)** | **Bias** | **Study design** | **Confounders** | **Blinding** | **Data Collection** | **Drop out** | **Global rating** |
| --- | --- | --- | --- | --- | --- | --- | --- |
| Pekrun (2019) | strong | moderate | strong | moderate | moderate | weak | moderate |
| Bonell (2018) | strong | strong | strong | strong | strong | strong | strong |
| Christiansen (2017) | strong | strong | strong | moderate | strong | strong | strong |
| Narhi (2017) | moderate | strong | weak | weak | weak | moderate | weak |
| Crooks (2016) | moderate | moderate | weak | moderate | weak | moderate | weak |
| Gonzales (2014) | moderate | strong | weak | moderate | strong | moderate | moderate |
| Jewett (2014) | moderate | moderate | weak | moderate | strong | moderate | moderate |
| Peltonen (2012) | weak | moderate | strong | weak | strong | moderate | weak |
| Stormshak (2010) | strong | strong | strong | moderate | strong | moderate | strong |
| Jorm (2010) | moderate | strong | strong | moderate | strong | strong | strong |
| Dodge (2007) | strong | strong | strong | moderate | strong | moderate | strong |
| Burke (1997) | strong | moderate | strong | moderate | strong | strong | strong |
| Gaston (2016) | moderate | strong | strong | strong | moderate | strong | strong |
| Velasquez (2015) | weak | moderate | strong | moderate | strong | strong | moderate |
| Komosa-Hawkins (2012) | strong | strong | strong | moderate | strong | strong | strong |
| Welkowitz (2000) | weak | moderate | weak | moderate | strong | strong | weak |
| Stoiber (2011) | moderate | strong | strong | moderate | strong | weak | moderate |
| Sawyer (2010a) | moderate | moderate | moderate | moderate | strong | moderate | moderate |
| Schmidt (2007) | moderate | moderate | strong | moderate | strong | strong | strong |
| Bonhauser (2005) | moderate | strong | strong | weak | strong | strong | moderate |
| Busch (2015) | weak | moderate | strong | moderate | moderate | moderate | weak |
| Cross (2018) | moderate | moderate | weak | moderate | moderate | moderate | moderate |
| Ford (2019) | moderate | strong | moderate | moderate | strong | moderate | strong |
| Fuscaldo (1998) | moderate | strong | moderate | moderate | weak | strong | moderate |
| Kamps (2000) | strong | moderate | strong | moderate | moderate | strong | strong |
| Sawyer (2010b) | moderate | moderate | weak | moderate | moderate | strong | moderate |
| Schachner (2016) | strong | strong | strong | moderate | strong | strong | strong |
| Bierman (2002) | strong | strong | strong | moderate | moderate | strong | strong |
| van den Berg (2018) | moderate | moderate | moderate | moderate | moderate | weak | weak |
| Warner (2018) | moderate | moderate | weak | moderate | weak | moderate | weak |
| Webster-Stratton (2008) | moderate | strong | strong | moderate | moderate | moderate | strong |
| Wienen (2018) | strong | moderate | NA | moderate | moderate | moderate | strong |
| Williford (2011) | moderate | moderate | moderate | weak | strong | weak | weak |
| Wright (2016) | moderate | moderate | strong | moderate | strong | strong | moderate |
| Green (2019) | weak | moderate | weak | weak | strong | weak | weak |
| Hawkins (2005) | moderate | strong | weak | weak | strong | strong | moderate |
| Ho (2017) | moderate | strong | strong | weak | strong | strong | moderate |
| Malakellis (2017) | moderate | moderate | weak | moderate | strong | weak | weak |
| Owens (2005) | moderate | moderate | weak | moderate | strong | weak | weak |
| Pfiffner (2016) | moderate | strong | moderate | weak | strong | strong | moderate |
| Young (2011) | strong | moderate | weak | moderate | strong | moderate | moderate |
| Dray (2017) | moderate | strong | strong | weak | strong | moderate | moderate |
| Herrera (2011) | weak | moderate | moderate | moderate | moderate | strong | moderate |
| McWilliams (1973) | moderate | moderate | weak | weak | weak | moderate | weak |
| Neal (2016) | weak | moderate | moderate | moderate | moderate | moderate | moderate |
| Newcomer (2016) | strong | weak | strong | weak | strong | moderate | weak |
| Rhie (2018) | moderate | moderate | moderate | moderate | weak | moderate | moderate |
| Bradshaw (2012) | moderate | weak | moderate | weak | strong | strong | weak |
| Karcher (2005) | moderate | weak | weak | moderate | strong | moderate | weak |
| Wahlstrom (2002) | weak | moderate | moderate | weak | weak | weak | weak |
| Dassanayake (2017) | weak | moderate | weak | weak | weak | weak | weak |
| Bierman (2007) | strong | strong | strong | weak | moderate | moderate | moderate |

**Table 3: Quality Assessment of Qualitative Studies**

| **1st author (year)** | **Research design appropriate to address the aims of the research** | **Recruitment strategy appropriate to the aims of the research** | **Data collected in a way that address the research issue** | **Relationship between researcher**  **and participants adequately considered** | **Ethical issues been taken into consideration** | **Data analysis**  **sufficiently rigorous** | **Research value** |
| --- | --- | --- | --- | --- | --- | --- | --- |
| Berg (2018) | Y | Y | Y | C/T | Y | Y | Moderate |
| Busch (2015)* | Y | Y | Y | C/T | Y | Y | Limited |
| Chawla (2014) | Y | Y | Y | N | Y | Y | Moderate |
| Crooks (2016)* | Y | C/T | Y | C/T | C/T | C/T | Moderate |
| Dassanayake (2017)* | C/T | N | C/T | N | N | C/T | Limited |
| Dunbar-Krige (2013) | C/T | C/T | Y | Y | N | C/T | Moderate |
| Hall (2010) | Y | N | Y | Y | N | C/T | Limited |
| Halsey (2005) | C/T | C/T | C/T | C/T | C/T | C/T | Limited |
| Hills (2016) | Y | C/T | Y | C/T | C/T | C/T | Moderate |
| Jalali (2018) | Y | C/T | Y | Y | N | Y | Limited |
| Kidger (2009) | Y | Y | Y | C/T | Y | Y | Moderate |
| Maller (2009) | N | Y | Y | C/T | N | N | Moderate |
| Michael (2013) | C/T | C/T | Y | N | Y | Y | Limited |
| Velásquez (2015)* | C/T | C/T | C/T | C/T | N | C/T | Limited |
| Wahlstrom (2002)* | N | C/T | N | C/T | C/T | C/T | Limited |
| Welkowitz (2000)* | C/T | C/T | C/T | C/T | N | C/T | Moderate |
| Zachariah (2018) | C/T | C/T | Y | N | C/T | C/T | Moderate |

 Notes: Y=Yes, N=No, C/T = Can't Tell. *mixed method studies

**Table 4: Findings**

| **1^st^ Author (year); country** | **Study Design** | **Study Aims** | **Intervention / factor grouping (physical, organisational, values, social/relational)**  **Description of the intervention (if present)** | | **Sample characteristics:  numbers of schools, number of participants at baseline/follow-up, intervention/control demographic characteristics** | **Setting primary/ secondary/both/ Pupil Referral Units (PRU)** | **Outcome measures** | **Key results** |
| --- | --- | --- | --- | --- | --- | --- | --- | --- |
| **Michael (2013); UK** | Qualitative | To investigate what young people who are attending PRUs due to exclusion from mainstream school perceive as enablers of and barriers to positive outcomes. | Multiple factors  No intervention | | 2 schools 16 participants at baseline & analysed  10 male 6 female  Key Stage 3 & 4 range 12-16 years | Pupil Referral Units | Semi-structured interviews asking about academic and socio-emotional positive outcomes. | Five themes representing enabling factors were identified: relationships (importance of teachers providing emotional support, positive relationship with teachers), curriculum (extracurricular activities, relevance/engagement and personalisation), discipline (effective and consistent sanctions), learning environment (size) and self.  Three themes representing barriers to the achievement of positive outcomes were identified: disruptive behaviour, unfair treatment (ineffective behaviour management strategies) and failure to individualise the learning environment (learning tasks not differentiated appropriately to suit individual learning needs).  Suggested changes included changing the appearance of the building, flexible and varied curriculum, feeling understood and listened to. |
| **Pillay (2013); UK** | Qualitative | To analyse and describe the reintegration experiences of learners with behavioural, emotional and social difficulties (BESD), followed by a resilience-based reintegration programme to aid policy makers and practitioners with the reintegration into mainstream education of learners with BESD. | | Multiple factors  No intervention | 4 schools 13 students completed qualitative interview  10 male 3 female 13 parents completed qualitative questionnaire 7 teachers completed email questions 3 education professionals completed qualitative interviews range 11-14 years | Pupil Referral Units | Unstructured interviews and questionnaires asking about promotive experiences and risk experiences across emotion, relationships and reintegration practices. | Learners experienced feelings of pride and optimism as promotive experiences during reintegration: - when their peers recognised their social progress - in themselves for developing social competence - in terms of academic competence and achievement  Emotions as a risk factor based on feelings of anxiety, anger and loneliness: - Experience of anger during the reintegration process  - Unconstructive peer relationships    Three significant categories of relationships were established: family relationships, peer relationships and relationships with adults in the educational setting.  Teacher participants indicated that the success of the reintegration process depended greatly on cooperation between the school and parents. The family was also experienced as a risk factor if learners experienced strained relationships within the family, a lack of academic support, low attachment to the family and variable family structure. Learners experience the reintegration process as a risk factor because of their disrupted academic progress, disengagement from the LSU/PRU and the disparity between the LSU/PRU and mainstream environment settings. |
| **Pekrun (2019): Germany (study 2)** | Cohort analytic | Testing a theoretical model linking achievement and emotions. | Organisational  No intervention | | 42 schools 1759 participants at baseline; 1759 analysed  Grades 5 & 6  50% female mean 11.7 years | Secondary schools | Achievement: PALMA Mathematical Achievement Test; PISA mathematics test  Emotions: Achievement Emotions Questionnaire-Mathematics  Self-concept: PALMA mathematics self-concept scale; PISA 2003 mathematics self-concept scale | Individual achievement positively predicted enjoyment and negatively predicted anger, anxiety, and hopelessness (p<0.05) Group-level achievement has a negative compositional effect on enjoyment and positive compositional effects on the negative emotions (p<0.05). These relations extend over the course of an entire school year. Self-concept might be a mediator both for the individual-level effects and the compositional effects of achievement on emotions. |
| **Bonell (2018); UK** | RCT | To test the impact of Learning Together intervention on self-reported bullying and perpetration of aggression and tests its cost-effective compared with standard school practice. | Multiple factors  Learning Together: All school staff were trained in restorative practices. Schools were provided with a manual to guide action group meetings to revise relevant school policies and coordinate the intervention. Lesson plans and slides were provided to guide teachers’ delivery of 5–10 hours per year of lessons on social and emotional skills for students in years 8–10 (age 12–15 years). School staff delivered primary restorative practices using respectful language to challenge or support behaviour and circle time to build relationships, and secondary restorative practices involved some staff implementing restorative conferences to address more serious behaviour problems. | | 40 schools (20 intervention, 20 control) 6667 participants at baseline (3320 intervention, 3347 control) Analysed at 24 months: 88·3% intervention, 90·4% control Analysed at 36 months: 81·2% intervention, 85·0% control  Year 8-10 range 11-15 years | Secondary schools | Gatehouse Bullying Scale (GBS), Edinburgh Study of Youth Transitions and Crime (ESYTC)  Paediatric Quality of Life Inventory (PedsQL), Short Warwick-Edinburgh Mental Well-Being Scale (SWEMWBS) Strengths and Difficulties Questionnaire (SDQ), Modified Aggression Scale (bullying subscale). | The intervention reduced students’ reports of bullying victimisation. Larger benefits were reported for secondary outcomes improved psychological functioning, wellbeing and quality if life, reduced police contact, smoking, alcohol and drug use. Adjusted results at 36 months: Adjusted difference, (95% Confidence Interval), p-value, adjusted effect GBS bullying score: –0·03, (–0·06, –0·001), 0·0441, –0·08 ESYTC: –0·13, (–0·43, 0·18), 0·4199, –0·03 PedsQL overall score: 1·44, (0·70, 2·17), 0·0001, 0·14 SDQ total difficulties score: –0·54, (–0·83, –0·25), 0·0002, –0·14 SWEMWBS total wellbeing index: 0·33, (0·00, 0·66), 0·0487, 0·07 Modified aggression scale: –0·26, (–0·57, 0·05), 0·0976, –0·12 |
| **Zachariah (2018); India** | Qualitative | To explore the impact of PE (Peer Educator) programs on the social, emotional, cognitive, behavioural, and attitudinal changes among voluntary PEs in the context of suicide prevention in India. | Social / Relational  Peer Educators programmes aim to empower students to respond to peers in distress and are widely used in suicide prevention. The main focus is interactions between peers in order to delineate the problem, encourage help-seeking and provide a safe space, and if needed, professional support.  In the context of suicide prevention PE training focused on improving knowledge of suicide warning signs and skills to respond to peers in distress, empathy, active listening, communication skills, non-judgemental thinking about others and increased self-care and awareness of own well-being. | | 6 schools 76 PEs involved for at least one school year completed an open-ended questionnaire Focus group session with 24 parents of PEs 3 Semi-structured interviews with school staff 10 Semi-structured interviews with PEs 4 Semi-structured interviews with program facilitators range 12-15 years | Secondary schools | Social, emotional, cognitive, behavioural, and attitudinal changes | No direct impact on MH.  Indirect and Unanticipated Results: Emotional awareness of PEs grew including the ability of students to introspect and explore their emotions, increased sense of mindfulness, increased feeling of self-worth, self-care value, particularly the value of their own life and how they live it, increased ownership and agency. Students became more independent, critical, and active in their approach to change negative aspects of their context.   The PE program had an impact on the PEs’ emotional development by way of increased emotional awareness and patience, feelings of self-worth, practice of self-care and building friendships. |
| **Christiansen (2017); Denmark** | RCT | To examine the effect of a 9-month school intervention focusing on physical self-worth, self-perceived sport competence, body attractiveness, social competences and global self-worth in children aged 10-13 years. | Organisational  The intervention was grounded in the Self Determination Theory. It consisted of initiatives targeting four settings for school-based physical activity: 1) PE classes, 2) in class activities, 3) break-time activities, and 4) theme days.  4th to 6th year teachers were given a tailored activity  programme, including educational materials, planning guides and PE lesson plans for incorporating PA throughout the school day. Teacher also participated in a competency development programme consisting of four full-day workshops focusing on the underlying theory and trying out core activities in practice. | | 24 schools (12 intervention, 12 control) 2916 participants at baseline (1349 intervention, 1567 control) 90% analysed in follow-up survey  Years 4-6  51.1% boys range 10-13 years  Family social class: 41% Upper-middle, 47% middle, 12% lower-middle | Primary schools | Physical self-worth: Children's Physical Self-Perception Profile Psychosocial self-perception and self-esteem: Self-Perception Profile for Children | Physical self-worth, self-perceived sports competence, body attractiveness, self-perceived social competence and global self-worth increased for both the intervention and control group during the 9-month follow-up.  Intervention effect (Baseline to follow-up between intervention and comparison group) Coef (95% CI) Physical self-worth: 0.02 (0.03, 0.07) Sports competence: 0.03 (-0.01, 0.08) Body attractiveness: 0.01 (-0.04, 0.07) Social competence: 0.02 (-0.03, 0.07) Global self-worth: 0.00 (-0.05, 0.05) |
| **Narhi (2017); Finland** | RCT | To evaluate the effects of a classroom intervention based on clear behavioural expectations, immediate positive feedback and weekly consequences for middle school classes with poor behavioural climate. | Social / Relational  Trainees were provided with the intervention manual and familiarised with the intervention and consultation procedures. One to two weeks after the beginning of the intervention; a consultation-based and focused on questions and troubleshooting in the implementation. In each school the intervention started with basic information on classroom management, especially on the importance of clearly articulated behavioural expectations and positive feedback for appropriate behaviour. Teachers discussed and agreed on the two most important student behaviours that disrupted the classroom behavioural climate of the participating class. The teachers were then instructed to rephrase the disruptive behaviours as behavioural instructions for the students. | | 35 schools (15 intervention, 20 control) Baseline 54 classes 54 teachers (25 intervention, 29 control). Mean number of students per class is 19.2 (SD 3.5, range 13-27) Follow-up 21 classes, 21 teachers intervention, 21 classes, 21 teacher control  Grades 7-8  55% male 45% female range: 12-13 years | Secondary schools | Self-report questionnaire for teachers and students developed by authors, asking about classroom behavioural climate. | Possibilities of concentrating on learning (i.e. ‘Students cannot work well’) in their classroom were significantly poorer than students in the PISA sample (representative sample of Finnish 15-year-old students in middle schools), but with negligible effect size [M = 2.07, SD = 0.59 and M = 1.94, SD = 0.78, respectively; independent samples t-test, t(1229.45) = 5.48; p < .001; Cohen’s d = 0.17]. No significant differences were observed on disruptive behaviour [‘There is noise and disorder’; present sample M = 2.48, SD = 0.74, PISA sample M = 2.50, SD = 0.82; independent samples t-test, t(1087.67) = 0.488; p = .325; Cohen’s d = 0.02].  The teachers’ evaluations of the classroom behavioural climate showed an improvement during the intervention, the improvement being larger and more consistent for the delayed control group having intervention in the spring term. The students’ evaluations showed intervention-related improvement only during intervention in the spring term. There were no intervention-related reductions in teacher-reported strain, although the effect in the spring term approached significance with a moderate effect size. The classroom behavioural climate stayed at a constant level from the post-intervention measure through the spring term for the classes that participated in the intervention in the autumn, and the follow-up indicated no deterioration in the behavioural climate next autumn. |
| **Crooks (2016); Canada** | Mixed methods (cohort analytic & qualitative) | To undertake a longitudinal evaluation of the effects of 1 or 2 years of program participation (Fourth R: Uniting Our Nations Mentoring Programs) on positive well-being, as assessed by mental health and cultural identity, across the transition from elementary to secondary school. | Social / Relational  The Fourth R: Uniting Our Nations Mentoring Programs is an evidence-based, healthy relationships and violence prevention intervention for schools. The programme, delivered by trained mentors is based on the Medicine Wheel life cycles. Beginning in the fall (West/Spiritual quadrant), sessions address interests, creating positive attitudes and atmospheres. The winter (North/Physical quadrant) sessions focus on bullying and healthy eating. The spring (East/Emotional quadrant) sessions focus on sharing, listening, goal setting and decision-making skills. Summer (South/Mental quadrant) look at communication skills, peer pressure, personal strengths and dealing with conflict. | | 61 schools (44 primary, 17 secondary) 139 participants at baseline analysed at wave 2 = 89%, at wave 3 = 85%, all three waves = 75.5%  93% First Nations  Grade 7-8  50.5 % male mean 12.6 years range 11-14 | Primary and secondary schools | Positive mental health: Mental Health Continuum—Short Form (MHC-SF) Cultural Identity: Cultural Connectedness Scale (CCS-Identity).  Qualitative findings include intra- and interpersonal changes, cultural learning and healthy relationships learning | Participants receiving 2 years of mentoring reported greater positive cultural identity [CCS-Identity; M(SD), 2 years of mentoring = 36.73 (3.66) compared to M(SD), 1 year or no mentoring = 33.21 (5.32); t(101) = -2.67, p = .009] and better mental health [MHC-SF; M(SD), 2 years of mentoring = 61.67 (11.51) compared to M(SD), 1 year or no mentoring = 52.03 (14.87); t(100) = -2.59, p = .011], as compared to those receiving 1 or no years of mentoring.  Qualitative findings: as the program progressed, students described improvement in peer relationship and building intimacy. The opportunity to build and strengthen bonds also extended to the relationships students developed with program facilitators.  Learning Culture served as a mechanism to establish an environment of trust and equality between students and facilitators. The program provided opportunities for students to connect their cultural teachings to their current life experiences, both in and outside of school.  Learning-Healthy Relationship Skills - youth discussed Fourth R communication and relationship strategies and articulated how they would apply their learning in real life situations. |
| **Gonzales (2016); USA** | RCT | To test effects of the Bridges to High School intervention on school engagement in 9th grade, following transition to high school, to examine whether 9th grade school engagement mediated intervention effects on academic, emotional, and behavioural outcomes in 12th grade. | Social / Relational  The intervention is a family-focused intervention to prevent MH and substance misuse disorders, and school dropouts amongst Mexican American adolescents attending schools in low-income, urban communities. The intervention includes: (a) a parenting intervention targeting parenting practices, child coping skills and family cohesion; (b) an adolescent coping intervention; and (c) a family strengthening intervention. | | 4 schools 516 participants at baseline analysed at T2 447 of 516 (86.6%), int 291 of 338 (86.1%), cont 156 of 178 (87.6%) analysed at T3 420 of 516 (81.4%); int 276 of 338 (81.7%), cont 144 of 178 (80.9%)  Grade 7-12  49.2% male mean: 12.3 years | Secondary school | Internal and externalising symptoms: Youth Self Report (YSR) and Adult Self Report (ASR)  High school drop-out: youth report, parent report, and school archival records  Substance use: 2001 Youth Risk Behaviour Survey Grade point average (GPA): Archival school data | Externalising: no significant direct or indirect effects for the intervention were found. Internalising: A significant indirect effect of the intervention on T3 internalizing through school engagement was found (ab = −0.166, 95% CI (−0.423, −0.001)) |
| **Jewett (2014); Canada** | Cohort analytic | To examine the association between participation in school sport during adolescence and mental health in early adulthood. | Organisational  No intervention | | 10 schools 880 participants at baseline; 853 analysed 7th grade  54% female  mean 20.4 | Secondary schools | Participation in school sport: self-report Depressive Symptoms: Major Depression Inventory (MDI) Perceived stress: questions designed by authors Self-rated mental health: questions designed by authors | School sport participation during adolescence was statistically significant predicator of lower depression symptoms (.36 (.17), .09*) [b (SE), β *p < .05], lower perceived stress) .05 (.02), .10*) and higher self-rated mental health (.05 (.02), .09*)  The effects were statistically significant after at least 5 years of participation. |
| **Peltonen (2012); Palestine** | Controlled trial | To examine the effectiveness of the School Mediation Intervention (SMI) in preventing mental health problems and promoting social functioning among children living in armed conflict. | Social / Relational  The School Mediation Intervention aims at enhancing conflict resolution and social dialogue and preventing disruptive school behavior. It focuses on: (a) introducing self-regulation techniques to attune provocative and aggressive behavior, (b) offering an atmosphere of safety and mastery for victims of peer aggression and bullying, and (c) enhancing attitudes, behavioral rules, and social conduct that improve school commitment and adjustment. | | 4 schools (2 int, 2 cont) 225 participants at at baseline (141 int, 84 cont)  172 analysed (76.5%) at T2  64% male  mean 11.37, range 10-14 years | Secondary schools | Depressive symptoms: Child Depression Inventory (CDI)  PTSD symptoms: Child Post Traumatic Stress Reaction Index (CPTS-RI) Psychological distress: Strengths and Difficulties Scale (SDQ) Prosocial behavior: Strength and Difficulties Scale (SDQ) Aggression**:** Multiple Aggression Questionnaire (MAQ) | Depression: symptoms increased in control and intervention groups, F(1, 149) = 5.21, p = .024, partial η2 = .03. Psychological distress: symptoms increased in control and intervention groups, F(1, 149) = 6.12, p = 0.14, partial η2 = .04. PTSD: Symptoms increased in intervention group, significant group main effect, F(1, 149) = 4.02, p = .047, partial η2 = .026, thus refers to T1 when intervention group had a lower level of PTSD symptoms. Intervention did not increase good friendship quality, prosocial behaviour and nonaggressive behaviour in the intervention group. |
| **Stormshak (2010); USA** | RCT | To examine outcomes associated with the Family Check-Up (FCU), an adaptive, tailored, family-centred intervention to enhance positive adjustment of middle school youth and prevent problem behaviour. | Social / Relational  The Family Check-Up intervention is delivered in middle schools resource centres. The aim is to engage and motivate parents to improve their parenting practices and use appropriate services to address their specific needs. It comprises of three brief sessions grounded in motivational interviewing. | | 3 schools  377 participants at baseline (277 int, 100 cont) analysed in Grade 7 = 329, 87% of sample; grade 8, n = 312, 83% of sample; grade 9, n = 289, 77% of sample  Ethnicity: White (36%), Latino/Hispanic (18%), African American (16%), Asian (8%), American Indian (3%), and biracial/mixed ethnicity (19%). Average household income of families 30-40k a year  6th grade | Secondary schools | Depression: 14-item questionnaire derived from items on the Child Depression Inventory  Self-Regulation: derived from the Early Adolescent Temperament Questionnaire School Engagement: questions devised by authors | Participation in the FCU was associated with increases in self-regulation from sixth to seventh grade (β = .08). Seventh grade self-regulation was associated with decreases in youths' depressive symptoms from sixth to eighth grade (β = −.25), with a small to medium effect size. Sixth grade levels of depression were not associated with changes in self-regulation by seventh grade. Contrary to prediction, eighth grade depressive symptoms were not associated with youths' ninth grade school engagement. However, eighth grade self-regulation was associated with a medium effect size increase in school engagement by ninth grade (β = .37). |
| **Jorm (2010); Australia** | RCT | To test if mental health first aid training improves teachers’ mental health knowledge, helping behaviours and confidence to help students, reduces stigma and improves knowledge of school policies and procedures. To test if trained teachers provide students with more information about mental health problems, which leads to improved students’ mental health. | Social / Relational  Teachers received a modified version of the Youth Mental Health First Aid course delivered over two day sessions, 7h each. The training covered departmental policy on mental health issues, common mental disorders in adolescents (depressive and anxiety disorders, suicidal thoughts and behaviours, and non-suicidal self-injury), and how to apply the mental health action plan to help a student with such a problem. Part two was specifically for teachers with responsibilities for student welfare. | | 14 schools (7 intervention, 7 control) Teachers: 327 baseline (221 intervention, 106 control)  Students: 1633 baseline (982 intervention; 651 control) Teachers: post-test questionnaire = 78%, follow-up questionnaire = 72% Students, follow-up questionnaire = 76% Years 8-10  46% male  range 12-15 years | Secondary schools | Teacher knowledge: questionnaire developed by authors | Students of the trained teachers were more likely to report that they received information about mental health problems (Odds Ratio)(OR) = 2.60, p < 0.001), including a “class lesson from teacher”, (OR = 2.76, p = 0.030), “poster, pamphlet, brochure or book” (OR = 4.84, p = 0.003) and “referral to website” (OR = 2.78, p = 0.045). The only other change was in one item measuring stigma perceived in others, with increases in the perception that others believe in unpredictability (OR = 1.64, p = 0.006). Contrary to the hypotheses, there was no difference in reported help received from teachers or in the students’ mental health. |
| **Bierman**  **(2007); USA** | RCT | To test the hypothesis that the Fast Track intervention would interact with risk level and would be more effective with highest risk children than moderate risk children. | Social / Relational  The intervention included parent behavior-management training, child social-cognitive skills training, reading tutoring, home visiting, mentoring, and a universal classroom curriculum.  During the elementary school phase of the intervention (grades 1-5), families received parent training with home visiting, academic tutoring, and social skills training. Parent and child group interventions were conducted during a 2-hour enrichment  programme. Group meeting were held weekly during grade 1 for 22 sessions, bi-weekly during grade 2 for 14 sessions, and monthly during grades 3 to 6 for 9 sessions each year. | | 54 schools 891 participants at baseline (445 int, 446 cont) Intention to treat analysis, all 891 included (lost to follow up: int = 80, cont = 102) Ethnicity: 51% African American, 47% European American, and 2% of other ethnicity  58% were from single-parent families, 29% of parents were high school dropouts, and 35% of the families were in the lowest socioeconomic class  69% male  mean 6.5 years | Primary schools | Psychiatric Criterion Counts and Disorders: Parent Interview version of the NIMH Diagnostic Interview Schedule for Children. Variables computed for conduct disorder (CD), oppositional defiant disorder (ODD), and attention-deficit/ hyperactivity disorder (ADHD) criteria Antisocial Behaviour: Self-Report of Delinquency instrument | Grade 3: For ODD, a significant interaction effect was found (coefficient -0.32, Standard Error (SE) 0.14; p < .03). For ADHD, a significant interaction effect indicated that the intervention reduced criterion counts as the severity of risk increased (coef -0.93, SE 0.35; p < .01). Grade 6: No significant main effects of intervention were found.  Grade 9: Antisocial Behaviour: Youths assigned to intervention received significantly lower ASB (coef -0.79, CI -1.4 to -0.2; p < .01). For CD, a significant interaction effect (coef -0.26, SE 0.09; p < .01), indicated lower scores for the highest risk intervention group than the control group. For ADHD, a significant interaction effect (coef -0.91, SE 0.31; p < .003) indicated lower scores for the highest risk intervention group than the control group. |
| **Burke (2010); USA** | Controlled trial | To investigate the effectiveness of socio-moral reasoning interventions in broad populations and the concurrent investigation of variables believed to be related to socio-moral development. | Social / Relational  The intervention was an element of a Life Skills course. It was delivered 3 days per week; each mentor worked with 3-4 middle school students.The goal of the mentors was to lead dilemma discussion groups and challenge the reasoning of their students, to form friendships with them, discuss issues raised by them and to serve as a role model. | | 1 school 528 participants at baseline (257 sixth-graders, 271 eighth-graders) 90% (297/328) analysed for the anxiety outcome (299 valid pre-test cases, 297 valid post-test cases, 297 cases with both valid pre- and post-test data).  Grades 6 & 8  Ethnicity: 55% European-American, 39% Hispanic, 4% African-American, and 2% of other origin.  52.5% male range 11-14 years | Secondary school | Socio-moral reasoning: Socio-moral Reflection Measure - Short Form Self-worth: Self-Perception Profile for Children Anxiety/Withdrawal subscale of the Child Rating Scale Empathic Concern and Personal Distress subscales of the Interpersonal Reactivity Index | Socio-moral reasoning scores: students who participated in the intervention would show superior gains in sociomoral reasoning scores over control group students.  For Anxiety/Withdrawal, the means consistently dropped across time periods from the beginning of the year to the end, with children reporting decreasing levels of anxiety. However, the differences were not large enough to meet post-hoc significance levels.  There was a significant difference between time periods one and two for Empathic Concern, with this measure of empathy dropping between time one and two, and rising mildly again in time three. |
| **Jalali (2018); UK** | Qualitative | To find out whether student perceptions of alternative provision change over the course of Primary to Secondary education. | Multiple factors  No intervention | | 3 schools 13 participants at baseline; 13 analysed  77% White British  Key Stages 2 & 4  85% male range 7-16 years | Pupil Referral Units | Semi-structured interviews asking about attribution of behavioural problems, supportive factors in PRUs, and reintegration into mainstream education | Attribution of behavioural problems:  External factors: being annoyed or targeted by peers, feeling unsupported and disliked by teachers or being unfairly blamed. Students viewing their behaviour as a form of protest to the treatment of others. Students also indicated a lack of understanding of the consequences of their behaviour, including their referral to the PRU. Students spoke of a sense of unfairness in how they were treated, reflecting the role of victim.  Supportive factors: the students highlighted many helpful aspects of the PRUs including having increased space and ‘calmness’, organisational structure (reward systems), increased support, the active and personalised nature of the learning curriculum.  Increased sense of control: Primary students associate this with the physical structure, whereas the secondary students increased adult support. Perceptions indicate that improved behaviour is still externally attributed to environmental factors, indicating a lack of change in thought patterns. |
| **Hills (2016); UK** | Qualitative | To build the capacity of schools to support the social, emotional and mental health needs of their pupils' using their own resources. | Social / Relational  No intervention | | 1 school 53 participants at baseline; 53 analysed  32 males range 4-11 years | Primary school | Questionnaires and semi-structured interviews asking about therapeutic relationships, dealing with feelings and building resilience | The importance of therapeutic relationship, exploring and understanding feelings, building resilience and teaching children to reframe situations were identified as key themes.  The children highlighted the importance of the ELSA teacher and how the relationship with this person was a key aspect relating to the effectiveness of the project. Children talked about the importance of being able to talk, discuss and understand their feelings and express / alter their feelings so that they could feel happier. Building resilience referred to reframing the situation, improving confidence and developing coping strategies. |
| **Gaston (2016); Canada** | Case control | To examine the effects of sitting on a stability ball (vs. regular classroom chair) on attention span, hyperactivity, oppositional defiant behaviours, and anxious/ depressive symptomatology among grade 2 students. | Physical  Sitting on a stability ball | | 1 school 44 participants at baseline (41 pupils [23 int, 18 cont], 3 teachers) 44 analysed  57% female  Grade 2 mean 7.2 years | Primary school | Inattention, Hyperactivity, oppositional-defiant behaviour, anxious/depressive symptomatology: NICHQ Vanderbilt (teacher) Assessment Scale Social validity: scale developed in previous study | Anxious/ Depressive symptomatology - Significant group differences emerged for 8-week follow-up scores (F (2, 41) = 9.25, p = 0.004, h2 = 0.20) but not for 5-month follow-up scores. Inattention (significant group difference): 8-week follow-up: (F (2, 41) = 15.34, p = 0.000, η2 = 0.29) and 5-month follow-up: (F (2, 41) = 4.80, p = 0.035, η2 = 0.11)  Anxious and depressive symptomatology (significant group differences at 8-weeks but not 5 months): 8-week follow-up (F (2, 41) = 9.25, p = 0.004, η2 = 0.20), 5-month follow-up (F (2, 41) = 0.14, p = 0.711, η2 = 0.00) Hyperactivity, Oppositional defiant behaviour: no significant differences. |
| **Velasquez (2015); Columbia** | Mixed methods (RCT & Qualitative) | To evaluate the impact of participating in extracurricular yoga workshops on the prevention of anxiety, depression, and aggression. | Organisational  After-school yoga workshops were delivered by an instructor trained in the Satyananda Yoga tradition. The children were taken through a number of postures, breathing exercises, relaxation and meditation techniques in each of the 24 2h sessions. | | 1 school 125 participants at baseline (68 int, 57 cont), 12 participated in focus groups 114 analysed  Grades 5, 8 and 9 | Primary and secondary school | Depression and anxiety: adapted from Strengths and Difficulties Questionnaire  Aggression: unlimited peer nomination procedure Socio-emotional competencies: scale items used in National Test of Citizenship Competencies | Results evidenced significant interactions for anxiety (F (1, 123) = 4.03; p < .05) and depression (F (1, 123) = 9.52; p < .05). Students who participated in the Yoga workshops reported a decrease in their anxiety and depression levels, while the control-group students experienced an increase in these indicators. Analyses with posttest scores as the dependent variable and pretest scores as the covariate showed no significant main effects of the programme on depression. There was a statistically significant decrease in anxiety (F (1, 123) = 3.87; p < .05), while no significant change in this variable was observed for the control group. According to Cohen’s d, children in the experiment group showed a reduction of .21 standard deviations in anxiety.  Qualitative findings – reports of stress reduction |
| **Komosa-Hawkins (2012); USA** | Controlled trial | To examine whether involvement in a school-based mentoring program targeting at-risk adolescents would lead to increases in social–emotional health. | Social / Relational  Mentors recruited from graduate programs in local universities were matched based on similar demographics and common interests with mentees from 9^th^ & 10^th^ grades. Matches met weekly once for 1 hour after school at the high school for one-to-one mentoring for the duration of 8 months. Mentors provided social support and engaged in discussions specific to educational and career planning. | | 1 school 43 participants at baseline (25 mentoring, 18 control) 43 analysed  Grades 9-10  Ethnicity: 48% African-American, 36% Latino, and 16% multiracial/other  80% of mentees were identified as being economically disadvantaged (free/reduced lunch recipients)  68% female mean 14 years 7 months | Secondary school | Social–emotional health: Behavioral and Emotional Rating Scale – 2nd edition Youth Rating Scale (BERS-2 YRS) | No significant main effect was found between the intervention and comparison groups, or over time, and there were no significant interactions. However, notable positive trends were found for five of six subscales (interpersonal strength, intrapersonal strength, school functioning, affective strength, career strength), with the intervention group showing greater positive changes as compared to the control group at post-intervention. |
| **Hall (2010); UK** | Qualitative | To explore if: (1) the Ten Element Map (MacDonald and O’Hara 1998) a useful framework to structure focus groups to elicit children’s views about organisational influences their mental health and wellbeing (2) information gathered from such focus groups can be used to develop an action plan for change at the organisational level (3) this process can promote pupil participation in the area of mental health and wellbeing? | Multiple factors  No intervention | | 1 school 18 participants at baseline; 18 analysed Reception - Year 6 | Primary school | Focus groups asking about quality of school environment, self-esteem, emotional processing, self-management, and social participation | Environmental factors with positive impact on wellbeing - outside areas and separate spaces outside, equipment and toys, clean and close toilets, clean tables in the dining hall, learning mentor’s room and a worry box.  Negative factors - food on the floor after lunchtime, concerns over safety, messy toilets and not enough space on the playground. Self-esteem - how other people in school helped them to feel good about themselves, adults let them know that they had done  Emotional processing - what happened when someone was upset: role that both the adults and the children had, telling an adult, if an adult does not know, it is not sorted’.  Self-management - what happens to help them look after themselves in school, physical aspects (e.g., putting plasters on when hurt), sense of humour helped them and others feel better (e.g., pulling funny faces and smiling), talking and solving problems helped. Social participation - what they enjoyed joining in with, after-school clubs, as well as equality of opportunity in school-based activities (‘Everyone should go on trips together’), humour and good relationships. |
| **Berg (2018); Canada** | Qualitative | To investigate participant perceptions toward the development and implementation of the student-led health inquiry initiative, and to extend social and emotional learning opportunities that foster resiliency. | Values  No intervention | | 4 schools 4368 students in the 4 schools, Got Health? Team consisted of 2-8 students leader and one teacher, exact numbers not reported.  Grades 7-12 range 12-17 years | Secondary schools | Focus groups asking about perception, awareness and stigmatisation of mental health, school culture, programme participation experiences | Three major themes emerged: Inspiration - raise mental health awareness, help improve the school environment for students and teachers, promote sense of community. Trials and tribulations - publicity, interest, traction and time. Gains - changed school culture concerning mental wellbeing. |
| **Dassanayake (2017); Canada** | Mixed methods (cohort analytic & qualitative) | To examine if building health promoting school environments by applying the CSH approach may decrease the anxiety and depression among students directly and indirectly. | Physical  No intervention | | 245 schools 75000 students school age pupils (ages not explicitly stated) | Not stated | Questions investigated the frequency in which the students experience feelings or display symptoms related to anxiety and depression scaled on a four-point scale: 0 = ‘Never or hardly ever’, 1 = ‘About once a week’, 2 = ‘About 2 or 3 times a week’ and 3 = ‘Every day or almost every day’.  Survey questions asking about common processes and strategies implemented by funded schools. | Quantitative findings - The schools that are in the actively-funded stage, relative to the ones in the pre-funded stage, had lower percentages of students with anxiety and depression. Specifically, the percentage of students that suffer from anxiety and depression were about 5 and 3% less in the actively-funded schools relative to pre-funded schools.  Qualitative findings – the five common processes and strategies that may have created the observed effects on anxiety and depression among actively-funded schools were:  Mental Health Initiative ranging from presentations and workshops for teachers, students and parents to increased training for teachers and support staff.  Education Initiatives such as healthy education sessions covering topics including mental health issues, healthy relationships  New Class Activities with a common strategy to increase student engagement.  Student Leadership Clubs in order to promote positive mental health.  Creation of ‘Healthy Spaces’ - The healthy spaces were created both inside and outside of the school buildings, and provided a space for students and teachers to take a healthy break to enjoy healthy snacks, movement breaks, and mental health breaks. |
| **Halsey (2005); UK** | Qualitative | To investigate the operational issues associated with Behaviour and Education Support Teams (BESTs) and to seek the views of other commentators including members of the BEST team and school personnel and explore the impact and effectiveness of BESTs. | Social / Relational  No intervention | | 29 schools Not stated Not stated range 5-18 years | Primary and secondary schools | Process evaluation: operational models used, developmental and sustainability issues, impact and effectiveness | Participants noted the impact of BESTs on self-esteem, confidence and overall happiness of children and young people. BEST support impacted on pupils’ ability to cope with the transition to secondary school, increasing their overall confidence about the move and thus reducing the risk of emotional and behavioural issues in Year 7. Given often complex needs of the young people they were working with, BEST and school staff had to recognise that the impact might be small overall, but small steps could make a big difference. While BEST could not always ‘solve’ problems, diagnosis of an underlying learning difficulty or facilitating a referral to another agency could be viewed as a successful outcome, leading to putting an appropriate support in place in the longer term. |
| **Welkowitz (2001); USA** | Mixed methods (controlled trial & qualitative) | To discuss activities and outcomes of the Mentor/Advisor Project, a program designed to promote positive self-concept and school success for high school students at risk of or experiencing emotional and behavioral disabilities in rural Vermont. | Social / Relational  Mentors/advisors were volunteers members of school faculty and staff. Mentors worked with 8 students at risk of emotional and behavioural difficulties. This included:  1) a Mentor/Advisor meeting regularly with a small heterogeneous group of students; 2) Community service learning projects developed and implemented by the students; 3) Personal Learning Plans developed by each student to explore an area of individual interest and their individual strengths; and, 4) a collaborative process for supporting the mentors. | | 1 school 63 participants at baseline N/A Grades 6-12  63.5% female  59% receiving free or reduced lunch | Secondary school | Emotional and behavioural strengths: Behavioural and Emotional Rating Scale (BERS) Life skills: collaborative teaming, personal goal planning, problem solving/decision-making, conflict resolution, community service, self-appraisal: scale developed by authors Self-reported impact of participation  Interviews asking about school climate, belonging, mastery, independence and generosity. | In intervention group there was a small but not statistically significant improvement in performance on certain indicators (i.e., days absent, disciplinary referrals) while students in the control group show a gradual trend towards less favourable performance. The control group started with significantly higher grades but there were no significant changes over time for either group. Both the mentor and control groups show similar declines on measures of perceived skill and school climate satisfaction over time.  Students in the "at-risk" group showed a decline in days absent over 4 semesters, while the non at-risk remained the same. Non at-risk group demonstrated a decline in performance on the skill survey over time, the at-risk did not demonstrate such a decline.  Student skill survey: Scores for the non-risk group went down over the course of one year, across time were significant at the 0.05 level.  No qualitative findings reported. |
| **Stoiber (2011); USA** | RCT | To determine the extent to which professional development was effective in bringing about positive change in educators’ (a) knowledge of functional assessment (FA) and positive support strategies, and (b) self-efficacy beliefs regarding accommodation of children with challenging behaviours. | Organisational  Teachers participated in professional development to better address the needs of children with challenging behaviours. The training included:  Phase 1: Teachers  nominated children for participation and completed behaviour rating scales for all children with parental consent. They also completed a self-assessment of their knowledge and skills related to FA and positive behaviour support (PBS) and self-efficacy beliefs about working with children with challenging behaviours  Phase 2: Teachers participated in one 5-hour training session and received procedural manual that included resource materials, record forms, training activities, and step-by-step procedures for implementing FA and PBS.  Phase 3: The second 5h training session focused on the process of establishing goals and writing benchmarks to monitor children’s progress toward goals.  Phase 4: Classroom observation of teacher’s and children’s’ practices conducted by trained observer over a 2-week period  Phase 5: Teachers completed FA for each child  Phase 5: Development of PBS plan  Phase 6: Implementation of PBS plan for 8-10 weeks | | 4 school districts 90 baseline 90 analysed  73% male  range 4-7 years | Primary school | Positive and challenging classroom behaviours: Social Competence Performance Checklist (SCP) | The intervention improved the social competent behaviour of children with challenging behaviour. Children in experimental classrooms showed significant within-group gains (RCI*>*1.96) in the occurrence of positive behaviours (based on SCP and BASC ratings), as well as significant reductions in behavioural challenges from pre- to post-intervention.  AT follow-up signiﬁcant differences (p < .001) between TARGET and CONTROL children, F (1, 63) = 20.02, η2 = .24, and three positive behaviour subscales: -- Self-Control F (1, 63) = 26.36, η2 = .30; -- Social Cooperation F (1, 63) = 22.05, η2 = .26; -- Learning Behaviour F (1, 63) = 22.12, η2 = .26.  - Follow-up tests showed signiﬁcant between-group differences F (1, 63) = 6.49, p < 0.01, η2 = .11, and three negative behaviour subscales: -- Aggression F (1, 63) = 10.33, η2 = .14; -- Noncompliance F (1, 63) = 10.62, η2 = .14; -- Negative Affect F (1, 63) = 12.53, η2 = .17. |
| **Sawyer (2010a); Australia** | RCT | To investigate the effectiveness of a universal intervention designed to reduce depressive symptoms among students commencing high school. | Multiple factors  Intervention consisted of 4 components: (i) Curriculum Intervention – aimed to improve problem solving and social skills, resilient thinking style and coping strategies; (ii) Building Supportive Environments – to improve the quality of social interactions amongst all members of the school community; (iii) Building Pathways for Care and Education – to facilitate adolescents’ access to support and professional services; and (iv) Community Forums – to provide young people, their families, and school personnel with information to assist them to identify problems, to seek help for themselves and to help peers. | | 50 schools (25 int, 25 cont) 5634 baseline (3037 int, 2597 cont) 5634 analysed  Year 8  47% male mean 13.1 years | Secondary Schools | Depression: Center for Epidemiological Studies Depression Scale (CES-D) Optimistic thinking style: scale devised by authors Interpersonal competence: modified version of the Adolescent Interpersonal Competence Questionnaire Coping actions: scale devised by authors Perceived social support: Multidimensional Scale of Perceived Support School climate: scale devised by authors | The linear time trajectory for CES-D scores indicated (coefficient = .517, SE = .222, p < .001) that average scores increased across the three assessments (i.e., baseline, 1-year and 2-year follow-up assessments). The coefficient for the group · time variable (coefficient = .052, SE = .309, p = .43) indicated that the linear trajectory of CES-D scores over time did not differ significantly between the intervention and comparison groups.  The linear growth trajectories across the three assessments indicated significant reductions in scores over time for Positive Coping Strategies (coefficient = .496, SE = .147, p < .001), Negative Coping Strategies (coefficient = .248, SE = .100, p < .001), Optimistic Thinking Style (coefficient = .716, SE = .115, p < .001), Family Relationships (coefficient = .838, SE = .068, p < .001), Friends Relationships (coefficient = .188, SE = .077, p < .001), and Significant Other Relationships (coefficient = .178, SE = .063, p < .001). |
| **Maller (2009); Australia** | Qualitative | To determine educators’ perceptions about the benefits of contact with nature for children’s mental, emotional and social health. | Physical | | 12 schools 35 baseline (28 lead teachers/principals, 7 from environmental education organisations) 35 analysed N/A | Primary schools | Interviews asking about educators’ perceptions about the benefits of contact with nature for children’s mental, emotional and social health. | The perceived benefits of unstructured activities for children’s mental, emotional and social health included: individual, self-directed experiences in nature as opposed to those activities where they are directed by adults, establishing a connection with nature as a result of their own discovery. Managers relayed the benefits of engaging in activities that give children a sense of empowerment and ownership, resulting in improved self-esteem. The empowerment arose from children experiencing responsibility and having the opportunity to use their initiative to solve environmental problems. School participants stated one of the main benefits from nature activities was an increase in children’s self-confidence through experiencing success and overcoming a physical challenge. |
| **Schmidt (2007); USA** | Controlled trial | To examine whether mentoring influenced children’s self-concept, anxiety, depression, and relationships with parents and peers over an 18-month period. | Social / Relational  College students served as mentors for local fourth graders for a period of two school years. Mentors visited with their children once a week at school and attended one Saturday event each month with the children and their families. | | 1 school 53 baseline 41 analysed  50% female median age starting 4th grade 9.67, ending 5th grade 11.25 | Primary schools | Self-concept: Piers-Harris Children’s Self-Concept Scale (PHCSCS) Anxiety: Revised Children’s Manifest Anxiety Scale (RCMAS) Depression: Children’s Depression Inventory—Short Form (CDI) Attachments: People in My Life measure (PIML) | Mentored children reported significant improvement over a period of 18 months in self-concept in the areas of behaviour (PHCSCS Behaviour p = .01) and anxiety (PHCSCS Anxiety p = .03), social concerns (RCMAS Social concerns p < .001) and worry (RCMAS Worry: p < .001). They reported marginal improvement in the self-concept scales of popularity and happiness, depression, and negative attachment with parents and peers. No significant changes in mentored children’s positive attachment with parents or peers. |
| **Bonhauser (2005); Chile** | Controlled trial | To evaluate the effects of a school-based physical activity program on physical fitness and mental health status of adolescents living in a low socioeconomic status area in Santiago, Chile. | Organisational  Three sessions of physical activity were held each week and each lasted 90 min. Each session consisted of three steps. The first step included minimum activity with no weight transfer. The second step included weight transfer activities and incorporated dynamic large muscle movements. The third step consisted of sports practice. | | 1 school 198 baseline 198 analysed  Grade 9  59% low income ($286 per month) mean 15 years | Secondary school | Anxiety and depressive symptoms: HADS (hospital anxiety depression scale) Self-esteem: Tennessee Self-Concept Scale | A statistically significant improvement of 13.7% in the anxiety score was observed in the group that participated in the program compared with a reduction of 2.8% in the control group (p < 0.001). The self-esteem score increased by 2.3% in the intervention group, but decreased by 0.1% in the control group (p< 0.001). No statistically significant difference in the depressive symptom score was observed between the groups. |
| **Busch (2015); The Netherlands** | Mixed methods (controlled trial & qualitative) | To evaluate the effects of a school-tailored Health Promoting School intervention, the Utrecht Healthy School, which integrated all five whole school characteristics. | Values  An intervention included creating healthy school policies, creating a healthy school environment (e.g., smoke-free school yard and a healthy school canteen), involve parents, teachers, and neighborhood in school changes and focus health education on skill and competency development. | | 4 schools T0 = 1716 T1 = 1692 T2 = 2393 T0 & T1 = 969 T0 & T2 = 605 all high-school years/grades | Secondary Schools | Psychosocial problems: Strengths and Difficulties Questionnaire (SDQ) Qualitative mapping of the implementation processes. | SDQ change in int v control in school A from T0 to T1 (B 1.27, p < 0.05) & from T0 to T2 (B 1.00, not significant). SDQ change in int v control in school B from T0 to T1 (B -0.12, ns) & from T0 to T2 (B 0.44, ns).  No insights on SDQ measure from the qualitative process evaluation were reported. |
| **Chawla (2014); USA** | Qualitative | To investigate how green schoolyards can reduce stress and promote protective factors for resilience in students. | Physical | | 6 schools 217 baseline 217 analysed  72/169 male  136 European-American  27 Hispanic  3 Asian  2 African-American  1 Pacific Islander  (info on 169 students only) range 6-19 years | Primary and secondary schools | How green schoolyards reduce stress and promote resilience | Natural areas enabled students to escape stress, focus, build competence, and form supportive social groups. The woods promoted cooperative alliances, autonomy, and competence. Parents also commented on the importance of the woods as a setting for physical and social competence, where children could explore and cooperatively create a fort culture of “work” with peers, away from expectations prescribed by adults. Students were able to protect their peace and calm because they could move freely through the habitat. Inside the building, students often engaged in arguments and rude, aggressive exchanges; but during more than 700 h of observations in the habitat, not a single incidence of such behaviour was seen. Teens gave four main reasons for the calm and peace that they felt during and after gardening: being outdoors in fresh air in nature; feeling connecting to a natural living system; caring for living things successfully; and having time for quiet self-reflection. Activities in the garden contrasted with sources of stress that students associated with other parts of school, including grades, homework, an enforced schedule, sitting for long periods inside, and pressure to win in competitive sports. |
| **Cross (2018); Australia** | RCT | To develop, implement and evaluate the Friendly Schools Project (FSP) intervention which aimed to reduce bullying and aggression among more than 3,000 students who had recently transitioned to secondary school. | Social / Relational  The FSP intervention involved three stages: i) Pre‐transition: Preparing for transition education booklets for parents and students; capacity‐building engagement with primary and secondary school staff (end of Grade 7). ii) Transition: Training and resources to support whole‐school and staff; classroom resources for Grade 8 students and newsletter tips for parents. iii) Post‐transition: Maintenance and resources for school staff to focus on Grade 9 student social competence, bystander and advocacy support. Comparison group received the usual transition, social and emotional and bullying prevention policies and practices as required by the Australian curriculum authorities. | | 21 schools 3068 baseline 2739 analysed  Grade 7-9 range 12-14 years | Secondary schools | Loneliness: seven items adapted from a 15‐item loneliness at school scale Depression, anxiety and stress: Depression Anxiety Stress Scale | Significant positive impact of the intervention was found at the first follow‐up data collection (end of Grade 8) on victimisation (p = 0.009), perpetration (p = 0.015), depression (0.017), anxiety (p = 005), stress (p = 0.036), loneliness (p = 0.007) and safety at school (p = 0.028). Controlling for differences at baseline, significant group effects were found for each dependent variable at first follow up. These impacts did not sustain to the second follow‐up at the end of Grade 9. |
| **Ford (2019); UK** | RCT | To evaluate the effectiveness and cost-effectiveness of the Incredible Years® Teacher Classroom Management (TCM) programme as a universal intervention, given schools’ important influence on child mental health. | Social / Relational  TCM was delivered to groups of teachers in six whole-day sessions. TCM’s explicit goals are to: enhance teacher classroom management skills and improve teacher–student relationships; assist teachers to develop effective proactive behaviour plans; encourage teachers to adopt and promote emotional regulation skills; and encourage teachers to strengthen positive teacher–parent relationships. This is accomplished through goal-setting, reflective learning, video-modelling, and role play, with cognitive and emotional self-regulation training. | | 80 schools Teacher: 2074, Parent: 1469, Child: 2054 baseline analysed at T1 (9 months): Teacher: 2001, Parent: 1291, Child: 1986 analysed at T2 (18 months): Teacher: 1848, Parent: 1229, Child:1888 analysed at T3 (30 months): Teacher: 1756, Parent: 1128, Child: 1760 Reception-Year 4  47% female  Ethnicity: 95% White, 0.6% Black, 1.1% Asian, 2.7% Mixed, 0.6% Other  12.2% eligible for free school meals  range 4-9 years mean 6 years | Primary schools | Mental health: Strength and Difficulties Questionnaire (teachers and parent versions) Classroom-based disruptive behaviour: Pupil Behaviour Questionnaire (PBQ) Children’s attitudes towards school: How I Feel About My School measure (HIFAMS) | Teacher-reported SDQ - not significant Total Difficulties score reduced by 1.0 point (95% CI 0.1, 1.9; p = 0.03) at the 9-month follow-up but did not persist at 18 and 30 months.  There was evidence, based on the PBQ score, of reduced disruptive behaviour across all three follow-ups (p = 0.04) and the Inattention/Overactivity score reduced (p = 0.02) across all waves. At 9 months, there was a reduction in peer relationship problems (p = 0.02) and an improvement in pro-social behaviour (p = 0.02). |
| **Fuscaldo (1998); USA** | Controlled trial | To report the results of a school-based programme for parenting teens. | Multiple factors  A comprehensive in-school intervention for adolescent mothers and their children involving parenting classes, parent support group, life-skills and job-skills training, tutoring of children by teachers and members of the community and mentoring of parents by positive role models. Comparison group was parenting teens that didn’t receive the intervention. | | 1 school 46 baseline 36 analysed at 2 years  Ethnicity: 93% Black, 4% Hispanic, 2% Other range 13-19 years mean 17 (at child’s birth) | Secondary Schools | Emotional stability and self-esteem: Brief Symptom Inventory; School Form of the Coopersmith Inventory  Parental stress: Parenting Stress Index | Intervention had a positive impact on the mean change in total positive symptoms of emotional health in program participants (3.21) vs comparisons (-1.81), p = 0.056, and general self-esteem PP (2.7) vs C (-0.69), p < 0.001. |
| **Kamps (2000); USA** | Controlled trial | This report is a further analysis of the prevention program investigation, including (a) findings from implementation across multiple school years with longitudinal student outcome data; (b) replication effects for the prevention program for the second cohort; (c) effects of variations in program implementation; specifically, strength of treatment and classroom structure; and (d) individual cases of treatment successes and failures. | Social / Relational  The intervention consisted of social skills activities, peer tutoring, and individual and classwide behavior management components. | | 4 schools 50 baseline 38 analysed at 4 years  92% male  Ethnicity: 71% African American, 24% European American, 5% Hispanic range 5- 11 years mean 8.2 years | Primary schools | Frequency of disruptive behaviours, ratings of appropriate classroom behaviour: Teacher Behaviour Report Form developed by authors | Direct observation data showed decreasing trends for aggression and out-of-seat behaviors. For Cohort 1, there were significant within-subject main effects for time of measurement and inappropriate behaviour with student aggression showing a significant decrease over time. Out-of-seat behavior showed slightly lower trends but no significant change. Negative verbal remarks to peers unfortunately showed increasing trends over time. For Cohort 2, moderate but nonsignificant decreases in aggression, out-of-seat behaviors, and negative verbals were noted. Increases in academic engagement and behavioral compliance were noted for both cohorts. Significant within-subject main effects for time of measurement and appropriate behaviors were noted for both cohorts, with improvements for engagement and compliance. |
| **Sawyer (2010b); Australia** | RCT | To investigate the effectiveness of a universal intervention designed to reduce depressive symptoms experienced by adolescents at high school. | Multiple factors  Intervention consisted of 4 components: (i) Curriculum Intervention – aimed to improve problem solving and social skills, resilient thinking style and coping strategies; (ii) Building Supportive Environments – to improve the quality of social interactions amongst all members of the school community; (iii) Building Pathways for Care and Education – to facilitate adolescents’ access to support and professional services; and (iv) Community Forums – to provide young people, their families, and school personnel with information to assist them to identify problems, to seek help for themselves and to help peers. | | 50 schools 5633 baseline 3512 analysed at 2 years  44% males  94% born in Australia, 3% indigenous background  In paid employment: 74% mothers, 93% fathers mean 13.1 years | Secondary schools | Depression: Center for Epidemiological Studies Depression Scale (CES-D) Optimistic thinking style: scale devised by authors Interpersonal competence: modified version of the Adolescent Interpersonal Competence Questionnaire Coping actions: scale devised by authors Perceived social support: Multidimensional Scale of Perceived Support School climate: scale devised by authors | CES-D (Depression): The linear time trajectory for CES-D scores (coefficient = .20, SE = .10, p < .05) indicated that average scores increased across the assessments. However, the group x time coefficient showed that the linear trajectory of CES-D scores over time did not differ significantly between the intervention and comparison groups (coef = -.19, SE = .19, p = .32). School climate (over 5-year period): For adolescent ratings, the coefficient describing the linear trajectory for School Climate scores (coef = -32, SE = .11, p < .01) was statistically significant demonstrating that, on average, adolescents’ sense of belonging and participation at school declined over time. For teacher ratings, the coefficient describing the linear trajectory of school climate was statistically significant (coef = .23, SE = .10, p < .05). The coefficient describing the group 3 time interaction was significant (coef = .43, SE = .20, p < .01). This indicates that teacher ratings of school climate increased for both groups, but the rate of increase for those in the intervention group was significantly higher than those in the comparison group. |
| **Schachner (2016); Germany** | Cohort analytic | The present study investigates the manifestation of two types of diversity policies (namely fostering equality and inclusion vs. valuing cultural pluralism) and the ethnic composition of the classroom, which are related to acculturation orientations and school adjustment among early adolescent immigrants. | Values | | 21 schools 574 baseline 396 analysed  54% female  93% born in Germany  Student origin: Turkey (38%), Italy (9%), Croatia, (5%), Other (48%)  23% of students attended a low vocational and 46% a medium vocational track, which allows an apprenticeship after graduation. A further 31% attended the high academic track, which allows university entry. mean 10.5 years | Secondary schools | Perceived equality and inclusion climate: adapted from the School Interracial Climate Scale  Perceived cultural pluralism climate: scale developed by authors Well-being: Self-Description Questionnaire for early adolescents, Satisfaction with Life Scale Psychological problems: items on depressive mood and physiological stress symptoms Behavioural problems: scales on delinquency and disruptive behaviour | Both types of climate (equity and inclusion and cultural pluralism) at T1 were associated with better student adjustment at T2. Perceived manifestation of diversity policies in the classroom improves immigrant students’ psychological school adjustment at the individual level and stronger orientation toward mainstream and ethnic cultures at the individual level and classroom. A smaller and more diverse immigrant group further promotes orientation toward the mainstream culture, at the classroom level. |
| **Bierman (2002); USA** | RCT | To describe the effects of the Fast Track program at the end of third grade for children assigned to the intervention condition compared to those in the control condition. | Multiple factors  The intervention consisted of high-risk children and their parents participating in a combination of social skills and anger-control training, academic tutoring, parent training, and home visiting. | | 54 schools 891 baseline (445 int, 446 cont) 871 analysed at 3 years (440 int, 431 cont)  51% African American, 47% European American, and 2% of other ethnicity (e.g., Pacific Islander and Hispanic)  69% male  58% single-parent families, 29% of the parents were high school dropouts, and 35% of the families were in the lowest socioeconomic class mean 6.5 years | Primary schools | Child Conduct Problems: TRF Externalizing T-score, TOCA-R Authority Acceptance score, Teacher Ratings of Child Behaviour Change, Parent Ratings of Child Behaviour Change (CPPRG). Aggression: peer nomination. Child Social Cognition: the Social Problem-Solving measure, Home Interview with Child. Child Social Competence: peer nomination. | Child Conduct Problems Five of the eight variables yielded significant main effects for intervention: TOCA-R Authority Acceptance (p < 0.01), Teacher Ratings of Child Behaviour Change (p < 0.001), Parent Ratings of Child Behaviour Change (p < 0.01), Parent Daily Report (p < 0.05), Special Education Diagnosis (p < 0.05)  Alternative approach to evaluating the impact of the intervention at the end of Grade 3: 37% of the children in the intervention group versus 27% of the control children were classified as problem-free. An analysis of covariance revealed a significant main effect of intervention, t = 2.91; p < .01, with an effect size of .21. |
| **van den Berg (2018); The Netherlands** | RCT | To investigate whether a careful rearrangement of the classroom seats could promote social acceptance and more prosocial behaviours for children with externalizing problems, and limit the potential negative consequences for classmates sitting next to them. | Social / Relational  The intervention consisted of identifying students high in prosocial behaviour and social status and seating them adjacent to students high in externalising behaviour. | | 28 schools (64 classrooms; 32 int [801 students], 32 cont [768 students] 1569 baseline 1435 analysed at 8-13 weeks  Grade 4-6  50% male  82% native Dutch, 3% first generation immigrant, 16% second generation immigrant mean 10 years | Primary schools | Likeability: peer report, scale derived by authors. Externalizing behaviour: teacher report, conduct problems subscale of the Strengths and Difficulties Questionnaire. Prosocial and aggressive behaviour: peer nomination. | Changes in target students' behaviour: - Teachers rated the target students as displaying fewer externalizing behaviours over time, (p = .02). - Target students who were initially disliked showed significantly fewer externalizing behaviours, according to teachers, over time in both conditions, (p = .03)  Prosocial behaviour: - Student rated: Regardless of condition, target students showed an increase in nominations for prosocial, F(1, 218) = 7.99, p < .01, ηp 2 = .04, and aggressive behaviour over time, (p < .01) - Liked target students were seen as more prosocial over time regardless of condition, F(1, 72) = 5.47, p = .02ηp2 = .07. Their aggressive behaviour did not change, F(1, 72) = 3.05, p = .09, ηp 2 = .04 |
| **Warner (2018); UK** | Controlled trial | To investigate whether a structured peer coaching programme in a sixth form environment can support students to increase their self-esteem whilst decreasing test anxiety and perceived stress. | Social / Relational  The intervention consisted of coaching skills training for trainers and trainees. Peer coaching then took place weekly. Sessions began with the coachee stating the issue they were looking to explore and then setting a goal in relation to this issue. They then explored the current reality of their situation, before developing a range of options for action and then finishing the session by looking at the steps and actions to take. | | 1 school 55 baseline (int = 27, cont = 28) 54 analysed (int = 26, cont = 28)  Year 13  54% male range 17-18 years mean 17 | Secondary school | Perceived Stress: Perceived Stress Scale Test anxiety: Westside Test Anxiety Scale Self-esteem: Rosenberg Self-esteem scale | Effect of intervention: - self-esteem: no main effect for condition (p = .168). - test anxiety: Significant main effect of condition (p = .016) with the coached condition having lower test anxiety than the control. - perceived stress: no significant main effect for condition (p = .199). |
| **Webster-Stratton (2008); USA** | RCT | To assess if training teachers to deliver the Incredible Years Dinosaur School Curriculum utilizing positive classroom behaviour management strategies would result in more positive and responsive teaching and less harsh or critical discipline, increased focus on social and emotional teaching, and more focus on parent involvement in children’s education than in control classrooms. | Social / Relational  The intervention consisted of the Incredible Years Teacher Classroom Management and Child Social and Emotion curriculum (Dinosaur School). Trained teachers offered the Dinosaur School curriculum to all their students in bi-weekly lessons throughout the year. They sent home weekly dinosaur homework to encourage parents’ involvement. Part of the curriculum involved promotion of lesson objectives through the teachers’ continual use of positive classroom management skills focused on building social competence and emotional self-regulation skills as well as decreasing conduct problems. | | 120 Head Start classrooms and 14 schools 1768 baseline in early Autumn 1746 analysed in late Spring  50% male  18% Latino, 18% African American, 20% Asian, 27% Caucasian, 8% African, 9% other minority  31% of the children did not speak English as their first language  Kindergarten-Grade 1 mean 5.3 years | Primary schools (& Head Start programs) | Observations of child conduct problems, emotional self-regulation and social competence: Multiple Option Observation System for Experimental Studies (MOOSES)  School Readiness and Conduct Problems: Coder Observation of Adaptation-Revised (COCA-R). General classroom atmosphere: Classroom Atmosphere Measure (CAS). | MOOSES: None of the 6 child MOOSES constructs (Child conduct problems, Child disengage, Peer involve, Child solitary, Child positive teacher, Child positive peer) showed significant main effects of intervention.  COCA-R: greater improvement in school readiness in the intervention than the control condition (p = 0.04) - larger impact on average student scores for teachers with students with very poor initial levels of school readiness. CAS: greater improvement in classroom atmosphere in the intervention than the control condition (effect size = 1.03, p-value = 0.0004). |
| **Wienen (2019); The Netherlands** | Cohort analytic | To evaluate whether the tier 1 SWPBS intervention, focusing on all children in the school, alters teacher perception of pupil behaviour and if the relative effectiveness differs across school types, teachers, and children. | Social / Relational | | 23 schools 16353 baseline 14110 analysed (at least 4 measurement points) not stated | Primary schools | Teacher perception of student behaviour: teacher-version of the Strengths and Difficulties questionnaire | All perceived problem behaviors showed a significant decrease over time, whereas prosocial behavior increased over time. Small effects sizes were present (Cohen’s d ~0.2).  For behavioral problems (s^2^ = 0.05; 95% CI, 0.04–0.08; p < 0.001), problems with peers (s^2^ = 0.06; 95% CI, 0.04– 0.09; p < 0.001), and prosocial behavior (s^2^ = 0.11; 95% CI, 0.08–0.16; p < 0.001) significant random slopes were found at the school level. |
| **Williford (2011); Finland** | RCT | To explore if there are mean level differences in the outcome measures between students receiving the KiVa Anti-bullying Program and those that are not and can reductions in peer-reported victimization predict improvement in students’ anxiety/depression levels as well as their peer perceptions. | Values  KiVa includes 20 h of curricula designed to increase anti-bullying attitudes in classrooms as well as defending behaviors and self-efficacy among bystanders. Lessons involve activities such as class discussions, group work, short films about bullying, role-playing exercises, and a five-level interactive computer game. KiVa also includes addressing identified cases of bullying. A team of three school staff members in each school works with the classroom teacher to resolve the issue through individual and group discussions with the victims and bullies. | | 78 schools NS 7741 students (3685 cont, 4056 int)  Grades 3-6  50.6% female  Most students were native Finns (i.e. Caucasian), with the proportion of immigrants being 2.1%. mean 11.2 years | Primary schools | Questionnaires asking about Depression: Beck Depression Inventory (BDI) Anxiety: combination of Fear of Negative Evaluation and the Social Avoidance and Distress scales | Students in the intervention condition reported significantly less victimization at wave 2 (latent d effect size = -1.08, p < 0.01) and wave 3 (-2.19, p < 0.01). Positive peer perceptions were equal at wave 1 (p = 0.46) but significantly different at wave 3 (p = 0.02) Depression: Roughly equal levels at waves 1 and 3 (p = 0.8 & p = 0.08) Anxiety showed decreases in both conditions over time, though a larger decrease was reported for those receiving the intervention; wave 1 (p = 0.26) wave 3 (p < 0.01). |
| **Wright (2016); USA** | Cohort analytic | To examine the buffering effect of adolescents’ perceptions of social support and technology mediation by their school resource officers at Time 2 (measured in seventh grade) in the associations among cyber victimization at Time 1 (measured in sixth grade) and psychosocial adjustment difficulties at Time 3 (measured in eight grade), including depression, anxiety, and loneliness. | Social / Relational | | 6 schools 977 baseline 867 analysed at 2 years  Grades 6-8  51% female  Ethnicity: 49% White, 30% Latino/a, 10% Black, 7% Asian, 4% biracial  Schools were from predominantly middle-class neighborhoods with 10-32% of students qualifying for free or reduced price lunch range: 13-15 years, mean: 13.67 | Secondary schools | Psychological adjustment difficulties: Center for Epidemiological Studies Depression Scale Anxiety: Multidimensional Anxiety Scale for Children Loneliness: Revised UCLA Loneliness Scale | Social support: second wave social support was related negatively to third-wave depression (B 0.26, p < 0.01) and anxiety, after controlling for the first and second waves of these variables. Second-wave social support moderated the association between first-wave cyber victimization and third-wave depression. In particular, the association between these variables was more negative at higher levels of social support, while such a relationship was more positive at lower levels of social support. No associations were found for third-wave anxiety and loneliness. In particular, the association between these variables was more negative at higher levels of social support, while such a relationship was more positive at lower levels of social support. No associations were found for third-wave anxiety and loneliness. |
| **Green (2019); Kenya** | RCT | To test if a school support intervention would lead to better mental health outcomes for orphans and conduct a mediation analysis to test the hypothesis that intervention effects on mental health are mediated by continued school enrolment. | Social / Relational  The intervention consisted of a) payment of school tuition fees for secondary school; (b) provision of a school uniform in primary school and the first year of secondary school; and (c) nurse visits. | | 26 (intervention 13, control 13) 837 baseline 835 (int 410, cont 425) analysed  Follow up at 2,3 & 4 years  Grade 7-11  48% female mean 14.8 years | Primary and secondary schools | Mental health: 5 items from Center for Epidemiologic Studies Depression Scale Revised (CESD-R) | Depression scores in the intervention group remained relatively stable throughout the 4-year period, while in control group they began to rise in year 2. By Year 4, the control group was worse on average compared to the intervention group.  The intervention does not seem to reduce symptom severity, but it seems to keep levels stable on average relative to the increase observed in the control group over time.  School support increased school enrolment and appeared to buffer against depression symptoms over time; this effect on depression might be mediated by the effect of the intervention on school enrolment. The total effect of the intervention on standardized depression scores at Year 4 was -0.28 standard deviations. The direct path was reduced by -0.02 (p = .10). Overall, 7.4% of the intervention effect on depression was mediated by continuous school enrolment, p = .10. This increases to 9.6% (p = .02) if we assume that students who were missing at follow-up were out of school. |
| **Hawkins (2005); USA** | RCT | To test whether an intervention administered universally in the elementary grades improved functioning in early adulthood, 9 years after the intervention ended. | Multiple factors  The intervention included the following 3 components: teacher training in proactive classroom management, interactive teaching, and cooperative learning, child social and emotional skill development, and parent training in child behavior management skills and in skills for supporting their children’s academic development delivered over 6 years in the full intervention group and over 2 years in the late-intervention group. Control group received no intervention. | | 18 schools 643 baseline 605 analysed  Follow up at 9 years  Ethnicity: 45% White American, 25% African American, 22% Asian American, 6% Native American, and 3% other ethnic groups.  56% of participants were eligible for the school free-lunch program  50% female Grades 1-6 | Primary schools | Emotional regulation: items assessing expressions of distress and anger devised by authors Mental health problems: Diagnostic Interview Schedule | Broad significant effects on functioning in school and work and on emotional and mental health were found. Fewer significant effects on crime and substance use were found at 21 years of age. Most outcomes had a consistent dose effect, with the strongest effects in subjects in the full-intervention group and effects in the late-intervention group between those in the full-intervention and control groups.  At 21 years of age, full-intervention participants reported significantly better regulation of emotions, compared with controls, as well as significantly fewer symptoms of social phobia and fewer thoughts about suicide. Effects of the full intervention on reducing symptoms and diagnostic criteria for depressive episode approached but did not achieve significance at p < .05. The late-intervention participants reported significantly fewer suicidal thoughts, and significantly fewer met diagnostic criteria for depressive episode at 21 years of age, compared with controls. No significant effects were found for either intervention group on generalized anxiety symptoms or diagnostic criteria or on social phobia diagnostic criteria. |
| **Ho (2017); Hong Kong** | RCT | To assess the effectiveness of a positive youth development (PYD) based sports mentorship program on the physical and mental well-being of adolescents recruited in a community setting. | Social / Relational  The sports mentorship was delivered by sports coaches to small groups of adolescents. The intervention was divided into 2 similar parts (each with 9 sessions). Each part started with sessions of introduction and warm up, during which the mentor introduced the chosen sport through deliberate play. Then, a half-session goal setting followed in which the students discussed what kind of sporting goal they would like to achieve. After setting the goals, the students spent 6.5 sessions building their sporting skills with the support of the mentors and peers, during which the mentors infused problem-solving techniques through experiential learning. Lastly, debriefing was conducted for skill consolidation and self-reflection. Students randomly assigned to the control group were provided with exclusive access to a Web-based health education game. | | 12 schools 692 baseline 664 (int 333, cont 331) analysed at 1 month  Birth place: 80% Hong Kong, 17% Mainland China, 2% Other  Maternal education: 43% lower secondary or lower, 43% upper secondary, 10% tertiary, 3% not disclosed  58% female mean 12.3 years | Secondary schools | Physical and mental well-being: Chinese version of SF-12v2 Psychological assets: self-efficacy and resilience, Chinese version of the Generalized Self-Efficacy Scale and the Connor Davidson Resilience Scale Social assets: Chinese version of the Resnick School and Family Connectedness Scales.‍ | The intervention improved students’ mental well-being (Cohen’s d, 0.25; 95% confidence interval [CI], 0.10 to 0.40; P = .001), self-efficacy (Cohen’s d, 0.22; 95% CI, 0.07 to 0.37; P = .01) and resilience (Cohen’s d, 0.19; 95% CI, 0.03 to 0.34; P = .02). |
| **Malakellis (2017); Australia** | Controlled trial | Assess a systems intervention (Australian Capital Territory ‘It’s Your Move!’ (ACT-IYM)) to prevent obesity among adolescents. | Values  The intervention consisted of multiple initiatives at individual, community, and school policy level to support healthier nutrition and physical activity. Components included: (i) increased collaborations with local food producers and Nutrition Australia to create healthy food policies; (ii) commitment to increasing healthy food consumption among staff and students; (iii) a focus on the relationship between health and food across all areas of the curriculum; (iv) traffic light colour coding of food sold at the school canteen by nutrition content. Each intervention school also had a unique objective that was specific to their identified needs and these were: School A: to increase the time adolescents spend in physical activity at school. School B: to significantly increase the proportion of adolescents using active transport to and from school who live within 30 minutes walking distance. School C: to increase mental wellbeing through the promotion of healthy eating and physical activity. | | 6 schools (3 intervention, 3 comparison) 880 baseline (int 628, cont 252) 656 analysed (int 499, cont 157) at 2 years range 12-16 years | Secondary schools | Mental health: mental wellbeing subsection of the Adolescent Behaviours, Attitudes and Knowledge Questionnaire (ABAKQ) Physical, emotional, social and school functioning, quality of life: Pediatric Quality of Life Inventory 4.0 (PedsQL) Depressive symptomatology: Short Mood and Feelings Questionnaire – Child Version (SMFQ), | Participants in School C reported a decrease (p<0.05) in depressive symptomatology between baseline (25.5%) and (17.4%) follow-up compared with an increase in the other two intervention schools from 16.3% at baseline to 20.2% follow-up. |
| **Owen (2005); USA** | RCT | To provide preliminary data to document the effectiveness of the Y.E.S.S. Program. | Multiple factors  Intervention consisted of a daily report card (DRC) procedure (on a daily basis, the teacher provided feedback to the child regarding the target behavior along with praise for achieving the goal, and the child delivered the DRC to the parents. Parents were encouraged to deliver a home-based privilege and consequence system contingent on the child’s performance), year-long teacher consultation regarding the child’s mental health problems behavior modification, behavioral interventions for the individual child, and classwide strategies, and parenting sessions tailored to the family’s needs emphasizing the importance of the home-based privilege system and frequent communication between home and school. Waitlist control children were recruited in the same manner as children in the treatment group; however, parents were informed that their child would be monitored for 1 year and would receive program services the following academic year. | | 2 schools 42 baseline (30 int, 12 cont) 42 analysed at 9 months mean 8 years | Primary schools | Diagnostic status ADHD, oppositional defiant disorder, conduct disorder: Disruptive Behavior Disorders (DBD) Rating Scale and the Impairment Rating Scale (IRS) Social, emotional, and behavioural problems: Child Behavior Checklist (CBCL) | [treatment coef, SE][waitlist coef, SE], comparison p-value], *p < .10, **p < .05  According to the parent report, treated children show marked reductions in hyperactive and impulsive (-0.12*, 0.05] [-0.10, 0.11], ns), oppositional or defiant ([-0.15*, 0.05] [0.09, 0.10], p < .05) and aggressive behavior ([-1.42*, 0.76] [1.39, 1.24], p < .10), and marked improvement in peer relationships ([-0.33*, 0.15] [0.16, 0.18], p < .06). Teachers observe treatment-related group differences in inattention ([0.01, 0.06] [0.06, 0.11] ns), academic functioning ([0.08, 0.24] [-0.05, 0.33], ns), and the student-teacher relationship. |
| **Pfiffner (2016); USA** | RCT | To evaluate the efficacy of the Collaborative Life Skills (CLS) psychosocial intervention. | Multiple factors  CLS is a 12-week intervention consisting of integrated school, parent, and student treatments delivered by school-based mental health providers. The classroom intervention consisted of a school– home daily report card, homework plan, and classroom accommodations as needed (e.g., preferential seating). Parent training included effective use of commands, rewards, and discipline, plus strategies (e.g., homework time, organization, independence in completing daily routines, peer interactions, and social skills) and stress management. Child social skills modules included good sportsmanship, accepting consequences, assertion, dealing with teasing, problem solving. Control condition received school and community services as usual. | | 24 schools 135 baseline 134 analysed  Grades 2-5  27% White, 10% Black, 23% Latino, 20% Asian, 20% multiracial  54.6% of students qualified for free or reduced lunch  Parent education: 61% college graduates  71% male mean 8.4 years | Primary schools | ADHD and ODD symptoms: Child Symptom Inventory (CSI) Cross-situational impairment (home and school): Parent and teacher Impairment Rating Scales Social Skills: Parent and teacher versions of the Social Skills Improvement System (SSIS) | Cohen’s d/Odds Ratio (95% CI), p value  *Significant after within-domain Benjamini-Hochberg false discovery rate correction  Students from schools assigned to CLS compared with those assigned to usual services had significantly greater improvement on parent (P) and teacher (T) ratings of ADHD symptom severity (P -1.05 (-1.42, -0.69), .0002*; T -0.67 (-1.02, -0.32), .0032*) and organizational functioning (P -1.09 (-1.46, -0.72), .0393*; T -0.68 (-1.03, -0.33), .0001*) and parent ratings of oppositional defiant disorder symptoms (-1.08 (-1.45, -0.71), .0002*) and social/interpersonal skills (0.39 (0.04, 0.74), .0034*). |
| **Young (2011); UK** | Cohort analytic | To explore if school context measured by pupil rating of school connectedness and perceptions of school environment is associated with rates of attempted suicide and suicide-risk at age 15 and self-harm at age 19. | Values | | 43 schools 2586 baseline  2196 at 4 years  1258 at 8 years mean (at baseline) 11 years | Secondary schools | Suicidality: attempted suicide, suicide risk and self-harm assessed in psychiatric interview Prior mental health risk: Kandel and Davies Depression Scale; use of psychiatric services | After adjustment for confounders, pupils attempted suicide, suicide-risk and self-harm were all more likely among pupils with low school engagement (15-18% increase in odds for each SD change in engagement). While holding Catholic religious beliefs was protective, attending a Catholic school was a risk factor for suicidal behaviours. This pattern was explained by religious ‘mismatch’: pupils of a different religion from their school were approximately 2-4 times more likely to attempt suicide, be a suicide-risk or self-harm. The only significant univariate association was that between (poor) overall school ethos and self-harm at age 19 (OR 1.45, p = 0.029). |
| **Dray (2017); Australia** | RCT | To evaluate the effectiveness of a universal, school based, pragmatic intervention targeting resilience protective factors in reducing four mental health problem outcomes. | Multiple factors  An intervention was developed to increase the provision of universal strategies targeting multiple internal and external resilience protective factors. Each strategy was designed to address one or more internal (cooperation/communication, empathy, goals/aspirations, problem solving, self-awareness, self-efficacy) or external resilience protective factor (school support, school meaningful participation, peer caring relationships). | | 32 schools 3115 baseline 2105 analysed at 3 years  50% male  Ethnicity: 76% White, 13% Aboriginal and/or Torres Strait Islander, 11% other ethnic, cultural or national origin  Socio-economic disadvantage: 57% most, 43% least  Grades 7-10  range 12-16 years | Secondary schools | Mental health: Strengths and Difficulties Questionnaire (SDQ)  Student internal and external resilience protective factors: Resilience and Youth Development Module of the California Healthy Kids Survey | Int vs. Control (Matched Cohort) [Mean difference (95% CI) p-value]  There were no significant differences between groups at follow-up for three mental health outcomes: total SDQ (0.47 (-0.41, 1.35) 0.27), internalising problems (0.05 (-0.54, 0.63) 0.87), and prosocial behaviour (-0.08 (-0.35, 0.19) 0.53). A small statistically significant difference in favour of the control group was found for externalising problems (0.43 (0.04, 0.83) 0.02). |
| **Herrera (2011); USA** | Controlled trial | To test the impacts of Big Brothers Big Sisters (BBBS) SBM on outcomes closely linked with the school context and on out-of-school outcomes. | Social / Relational  Mentors from local businesses and high schools and in some cases, from colleges. Mentors received 1h of training and ask to meet their mentees at least once weekly. Meetings with mentees (Littles) involved engaging in tutoring or homework help and a wide variety of other activities, including creative activities (e.g., drawing, arts and crafts), games and discussions about various issues and topics. | | 71 schools 1139 baseline analysed at 9 months: 1067 analysed at 15 months: 968  23% Latinos, 18% African Americans, 13% multiracial youth  69% of pupils receive free or reduced-priced lunch  54% female  Grades 4-9 range 8-18 years, mean 11.2 | Primary and secondary schools | Social Acceptance: six-item subscale of the Self-Perception Profile for Children Teacher relationship quality: short version of the Student-Teacher Relationship Scale Parent relationship quality: seven items from the Parent Trust subscale of the Inventory of Parent and Peer Attachment Global Self-Worth: eight-item subscale of the Self-Esteem Questionnaire Stressful Life Events: adapted from the Social Readjustment Rating Scale | 9 months: Littles (treatment group) teachers reported significantly (p < .05) better overall academic performance and the Littles themselves reported more positive perceptions of their own academic abilities (p < .05) than their non-mentored peers.  15 months: No significant differences between Littles and their non-mentored peers on any of the 11 outcome measures. In general, across the youth outcomes assessed, Littles improved more than their non-mentored peers by the end of the first school year but then declined to levels equivalent to those of their non-mentored peers by late fall of the second school year. |
| **Kidger (2012); UK** | Qualitative | To examine the views of students and staff involved in emotional health work regarding current school-based emotional health provision, how far this meets the needs they identify and what they would like to see schools do in the future. | Multiple factors | | 8 schools qualitative student focus groups (27 groups, 154 students) and staff interviews (12 interviews, 15 individuals)  51% below average free school meal eligibility  Years 8-9 range 12-14 years | Secondary schools | Emotional health in the curriculum, support for those in distress, and the physical and psychosocial environment. | The school environment findings from staff interviews and student focus groups highlighted the importance of the school environment, both physical and psychosocial, in affecting students’ emotional health. Key themes that emerged were the sources of distress that existed in the school context: bullying, difficult relationships with teachers and academic work. Schools could bolster students’ emotional health through whole-school changes: fostering a supportive culture and improving the physical environment.  Support for students in distress - The importance of having someone to talk to, a lack of confidential, accessible and sympathetic help sources within schools, the importance of having easily accessible safe spaces within school to work through emotions, and the need to find non-stigmatising ways of providing support. |
| **McWilliams (1973); USA** | Controlled trial | To evaluate the effectiveness of a programme that involves selected high school students who were trained as mental health aides and worked with primary grade children referred for school maladaptation problems. | Social / Relational  High schoolers were trained in the basic concepts of psychological adjustment, children’ s problems and feelings and specific activities they might employ during mentoring. Contact sessions lasted from 25 to 30 minutes and occurred during aides' study halls or free periods. In addition to relation-building activities such as conversation, walking, etc., typical activities during sessions included drawing, painting, crafts, indoor and outdoor games, and reading. | | 2 schools 50 baseline 50 analysed Not clearly reported. Aides: sophomore, junior and senior grades Elementary children: Kindergarten - third grade | Primary and secondary schools | Behaviour: AML Behavior Rating Scale (teacher rating); Child Behavior Change Scale (aide rating) | Mean changes scores, t ratios testing the significance of differences between change between groups for AML (*p < .05, **p < .01 one tailed):  Experimentals (E) improved significantly more than controls (C) on the "Is unhappy or depressed" item (E 4.7, C 4.04, 2.23*) as well as the sum acting-out behavior (E 20.87, C 18.96, 1.92*) and overall sum behavior rating (E 23.26, C 19.41, 1.91*). Aides perceived children as becoming significantly more warm (1.04, 4.93**), trusting (.65, 3.82**), friendly (1.17, 1.47**) , and attentive (.65, 3.61**); less withdrawn (-1.0, -4.35**), sad (-.94, -4.95**) and frightened (-1.58, -4.65**). Additionally, they rated children as significantly improved in terms of behavioral (1.0, 4.0**), educational (.92, 3.17**) and overall change (1.34, 6.09**). |
| **Neal (2016); UK** | Cohort analytic | To identify which universal intervention strategies employed by primary schools were associated with children's post-transition anxiety, whilst controlling for their anxiety before the move. | Social / Relational | | 9 schools NS 621 analysed at 6 months  12.6% eligible for free-school meals 37% minority ethnic background  50% male mean 11.2 years | Primary and secondary schools | Generalized and school anxiety: pupil reports on the school and generalized anxiety subscales from the Screen for Child Anxiety Related Emotional Disorders (SCARED) | The addition of transition strategies and SEN group at step two did not significantly improve either model (School Anxiety: R^2^ Change = .007, p = .22; Generalized Anxiety: R^2^ Change, p .003, p = .63). However, inspection of individual predictors in model 1 (school anxiety) highlighted a main effect of systemic strategies (beta (b) = -.09, p = .03), indicating that these were associated with lower school anxiety (adjusting for prior school anxiety). No significant main effect was found for cognitive (b = .03, p = .53) or behavioral (b = .01, p = .77) strategies. Similarly, no strategies were associated with generalized anxiety at T2 (Cognitive strategies: b = -.01, p = .80; Behavioral strategies: b = .02, p = .64; Systemic strategies: b = -.05, p = .17). SEN specific strategies were not associated with school (b = .04, p = .83) or generalized (b = -.23, p ¼ .19) anxiety within the SEN population. |
| **Newcomer (2016); USA** | Mixed methods (RCT & cohort analytic) | To explore whether first and second grade peer social preference mediates the Good Behavior Game’s (GBG) effect on risk for future suicide attempts. | Social / Relational  The GBG teachers initially received training. The teacher makes explicit classroom rules of student behavior and teams were rewarded if the team members committed four or fewer infractions of these classroom rules. Over time, the game was played at different times of the day and during different activities. In this manner, the GBG evolved from a procedure that was highly predictable and visible, with a number of immediate rewards, to a procedure with an unpredictable occurrence and location, with deferred rewards. Control group were in the standard school program. | | 19 schools 2311 baseline 1385 analysed at 2 years  60% male  90% Black  60% reduced/free meal status Grades 1-2  mean 9.3 | Primary schools | Social preference: peer nomination Aggressive, disruptive behaviour: Authority Acceptance Scale of the Teacher Observation of Classroom Adaptation – Revised (TOCA-R) Depressive symptoms: Child Depression Index (CDI) Suicide attempts: Affective Disorders module of the National Institute of Mental Health Diagnostic Interview Schedule for Children Version 2.3 (NIMH DISC-2.3) | Results indicated that peer social preference partially mediated the relationship between the GBG and the associated reduction of risk for later suicide attempts by adulthood, specifically among children characterized by their first grade teacher as highly aggressive, disruptive. |
| **Rhie (2018); South Korea** | Cohort analytic | To confirm the effects of delayed school start time on sleep duration, school performance, mood, and sleepiness. | Organisational  Intervention group began the school day at 9am. Control group continued to start the school day from 7.30am-8am. | | 800 schools NA (secondary data) 42,517 (int group) 28,287 (cont group)  51% male Grades 7-11 | Secondary schools | Depressive mood, suicidal ideation, stress level: Korea Youth Risk Behavior Web-based Survey (KYRBS) | Subjective happiness improved and stress levels decreased for students in both intervention and control groups. Depression levels during the past 12 months and suicidal thoughts and plans for past 12 months decreased in both groups.  Degree of fatigue recovered from sleep for the past 7 days in the treatment group was rated as very sufficient and sufficient temporarily improved and returned to that of the pre-campaign, while the ratings in the control group did not change by year. |
| **Bradshaw (2012); USA** | RCT | To examine the hypothesis that children in schools implementing School-Wide Positive Behavioral Interventions and Supports (SWPBIS) would have better teacher-rated emotion regulation and prosocial behaviours and fewer concentration problems and disruptive behaviours. | Social / Relational  SWPBIS is a noncurricular universal prevention strategy that aims to alter the school’s organizational context to implement enhanced procedures and systems to guide data-based decisions related to student behavior problems and academics. Schools establish a set of positively stated, school-wide expectations for student behavior, which are taught to all students and staff. The comparison schools refrained from implementing SWPBIS for 4 years. | | 37 schools 12835 baseline 11738 analysed at 4 years  Ethnicity: 45% African American, 46% Caucasian, 4% Hispanic, 4% Asian/Pacific Islander, 1% American Indian/Alaskan Indian  49% receive free or reduced priced meals  53% male Kindergarten-Grade 2 | Primary schools | Aggressive and disruptive behaviours: Teacher Observation of Classroom Adaptation - Checklist (TOCA-C) | The multilevel analyses indicated a significant positive intervention effect on disruptive behaviours (p < .05, effect size (ES) = 0.12), concentration problems (p < .05, ES = 0.08), prosocial behaviour (p < .05, ES = –0.17 and emotion regulation (p < .05, ES = –0.11).  Children who were in kindergarten when the trial began fared better in SWPBIS schools than in comparison schools on both prosocial behaviour (p < .01) and emotion regulation (p < .05). Children in SWPBIS schools were 33% less likely to receive an ODR than those in the comparison schools (p < .001) |
| **Karcher (2005); USA** | RCT | To examine the effect of mentors’ attendance on their mentees’ outcomes after six months of developmental mentoring. | Social / Relational  Mentoring was conducted one-on-one in a group format twice weekly after school for two hours at the middle school. Mentees and mentors self-selected each other after a six-hour Saturday orientation. The sequence of daily after-school activities included an “icebreaker,” a connectedness curriculum activity, a snack, and a group game or recreational activity (e.g., playing tag, doing artwork, playing basketball). . The mentees and comparison group only differed on mean number of academic risks. | | 1 school 33 baseline 24 analysed at 6 months  63% male  Mentees: Grades 4-5 Mentors: Grades 6-8 | Primary and secondary schools | Teacher-rated risk status: developed by authors, includes items on inadequate social skills, few friends, emotional/behavioural problems, difficulty getting along with others, and problems with authority Adolescent connectedness: Hemingway: Measure of Preadolescent Connectedness (Version 3) Self-esteem: Harter Self-Perception Scale for Children Social and school competence: Primary Mental Health Project (PMHP) Child Rating Scale | An overall positive effect of program participation on connectedness to school and parents was found, but the hypothesized mediation model was not supported. Main effect of mentoring on connectedness to school and to parents.  Mentor attendance was positively related to changes in mentees’ self-reported rule compliance (self-management), social skills, and self-esteem, specifically in self-perceptions of attractiveness. Mentor attendance also related positively to the total connectedness change score, but not the connectedness to school or parents change score. |
| **Wahistrom (2002); USA** | Mixed methods (cohort analytic & qualitative) | To explore findings from a 4-year study in a large, urban school district that altered high school start times significantly from 7:15 a.m. to 8:40 a.m. and whether the positive outcomes that were evident during the first year of the change were persisting over the long term. | Organisational | | 7 schools NA (secondary data) 50962 analysed Grades 9–12 | Secondary schools | Feelings and behaviours associated with depression: items from the School Sleep Habits Survey Self-reported impact of delayed start time | The students whose high schools started at 8:30 a.m. or later reported significantly less depressive feelings than did the early start students (mean 8:40 start time = 10.37 (p < .05) to mean 7:30 start time = 10.89 (p < .05).  Qualitative findings - students in the intervention group that started school at 8:30am or later reported increased stress. |
